# CRISPR screening in human trophoblast stem cells reveals both shared and distinct aspects of human and mouse placental development

**DOI:** 10.1073/pnas.2311372120

**Published:** 2023-12-12

**Authors:** Takanori Shimizu, Akira Oike, Eri H. Kobayashi, Asato Sekiya, Norio Kobayashi, Shun Shibata, Hirotaka Hamada, Masatoshi Saito, Nobuo Yaegashi, Mikita Suyama, Takahiro Arima, Hiroaki Okae

**Affiliations:** ^a^Department of Informative Genetics, Environment and Genome Research Center, Tohoku University Graduate School of Medicine, Sendai 980-8575, Japan; ^b^Department of Obstetrics and Gynecology, Tohoku University Graduate School of Medicine, Sendai 980-8575, Japan; ^c^Department of Trophoblast Research, Institute of Molecular Embryology and Genetics, Kumamoto University, Kumamoto 860-0811, Japan; ^d^Department of Mechanical Engineering, University of Michigan, Ann Arbor, MI 48109; ^e^Division of Bioinformatics, Medical Institute of Bioregulation, Kyushu University, Fukuoka 812-8582, Japan

**Keywords:** human trophoblast stem cells (hTSCs), CRISPR screening, placental development, transcription factor

## Abstract

The placenta serves as the interface between the mother and fetus, facilitating the exchange of gases and nutrients between their separate blood circulation systems. Trophoblasts in the placenta play a central role in this process. Our current understanding of mammalian trophoblast development relies largely on mouse models. However, given the diversification of mammalian placentas, findings from the mouse placenta cannot be readily extrapolated to other mammalian species, including humans. To fill this knowledge gap, we performed CRISPR knockout screening in human trophoblast stem cells (hTSCs). We targeted genes essential for mouse placental development and identified more than 100 genes as critical regulators in both human hTSCs and mouse placentas. Among them, we further characterized in detail two transcription factors, DLX3 and GCM1, and revealed their essential roles in hTSC differentiation. Moreover, a gene function–based comparison between human and mouse trophoblast subtypes suggests that their relationship may differ significantly from previous assumptions based on tissue localization or cellular function. Notably, our data reveal that hTSCs may not be analogous to mouse TSCs or the extraembryonic ectoderm (ExE) in which in vivo TSCs reside. Instead, hTSCs may be analogous to progenitor cells in the mouse ectoplacental cone and chorion. This finding is consistent with the absence of ExE-like structures during human placental development. Our data not only deepen our understanding of human trophoblast development but also facilitate cross-species comparison of mammalian placentas.

## Significance

The survival and growth of the mammalian fetus depend on the placenta, with trophoblasts playing essential roles in this organ. The molecular mechanisms underlying mouse trophoblast development have been intensively analyzed. However, the extent to which findings from mouse models are applicable to humans remains uncertain. Here, we applied CRISPR screening to human trophoblast stem cells and identified numerous genes that are essential for human trophoblast proliferation and differentiation. Notably, we identified two transcription factors, DLX3 and GCM1, as key regulators of human trophoblast differentiation. Moreover, we carefully compared the results of our CRISPR screening with the phenotypes of previously reported mutant mouse strains, which provides valuable insights regarding the analogies between human and mouse trophoblast subtypes.

Trophoblasts in the placenta play pivotal roles in gas and nutrient exchange, hormone production, fetal anchorage to the uterine wall, and fetal protection from the maternal immune system (1). While the placenta is well conserved among placental mammals and is essential for embryonic development, it is also one of the most rapidly evolving organs (2, 3). The molecular mechanisms underlying mammalian placental development have been intensively analyzed in mice. However, owing to the diversification of mammalian placentas, findings from the mouse placenta cannot be readily extrapolated to other mammalian species, including humans (4, 5). The molecular mechanisms regulating human trophoblast development have only recently begun to be evaluated in detail owing to the prior lack of suitable model systems.

In 1998, mouse trophoblast stem cells (mTSCs) were derived from blastocysts and the extraembryonic ectoderm (ExE) of postimplantation embryos, opening the door to in vitro investigation of mouse trophoblast development (6). In 2018, our group reported the successful establishment of human trophoblast stem cells (hTSCs) from blastocysts and cytotrophoblasts (CTs) isolated from first-trimester placentas (7). hTSCs are highly proliferative and have the potential to differentiate into two differentiated trophoblast lineages, syncytiotrophoblasts (STs) and extravillous cytotrophoblasts (EVTs). Recent single-cell RNA-Sequencing (scRNA-Seq) analyses have shown that hTSCs are most similar to peri- or post-implantation CTs (8, 9). Shortly after the establishment of hTSCs, two groups developed culture systems for human trophoblast organoids (10, 11). Subsequently, hTSCs and trophoblast organoids have become widely used as in vitro models to investigate human trophoblast development and function.

In both humans and mice, trophoblast cells originate from the trophectoderm (TE), the outer layer of the blastocyst, and TSCs have been established. However, the culture conditions of hTSCs and mTSCs differ substantially (6, 7). Moreover, it is unclear which human and mouse trophoblasts are analogous. To fill this knowledge gap, in this study, we performed CRISPR screening of selected genes in hTSCs and compared the results with the phenotypes of previously reported knockout (KO) mouse strains. Our analyses suggest that hTSCs may be analogous to progenitor cells in the ectoplacental cone (EPC) and chorion but not to stem cells in the ExE. Moreover, we identified two transcription factors (TFs), GCM1 and DLX3, as essential regulators of EVT and ST differentiation. In contrast, these TFs only regulate labyrinth development in mice (12, 13). These findings are fundamental for understanding the mechanisms underlying human trophoblast development and provide valuable insights regarding the analogies between human and mouse trophoblast subtypes.

## Results

### CRISPR Screening in hTSCs.

**Fig. 1.**
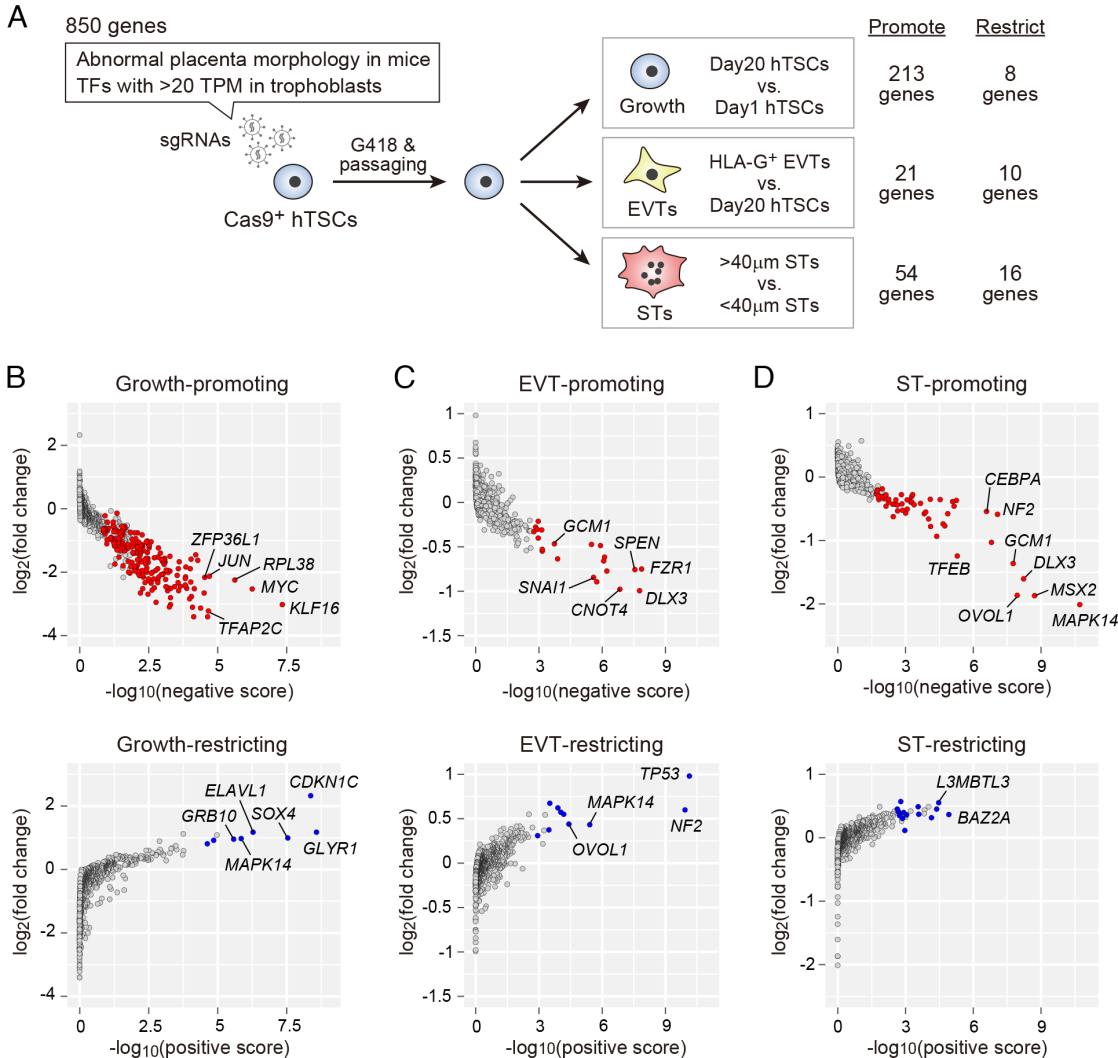
CRISPR screening in hTSCs. (*A*) Schematic representation of our CRISPR screening strategy. sgRNAs targeting a total of 850 genes were transfected into Cas9-expressing hTSCs. After selecting sgRNA-expressing cells with G418, these cells were maintained or differentiated. We used an anti-HLA-G antibody to isolate EVTs and performed a size selection to separate STs (>40 µm) from poorly fused (<40 µm) cells. The numbers of genes classified as essential for hTSC growth or differentiation (false discovery rate (FDR) < 0.05) are indicated. (*B*) Identification of genes required for hTSC growth. Negative and positive scores and fold changes were calculated using MAGeCK (14). Statistically significant growth-promoting and -restricting genes (FDR < 0.05) are shown in red and blue, respectively. Several representative genes with low FDR values are indicated. (*C*) Identification of genes required for EVT differentiation. (*D*) Identification of genes required for ST differentiation.

To select appropriate genes for CRISPR screening, we searched the MGI database and focused on 426 human genes that are orthologs of mouse genes associated with “abnormal placenta morphology.” Considering the critical roles of TFs in stem cell maintenance and differentiation, we also included TFs with intermediate or high expression levels in primary human trophoblasts or hTSCs [>20 transcripts per kilobase million (TPM) in at least one cell type, based on our previous study (7)]. This resulted in the selection of 850 genes (Fig. 1*A* and Dataset S1). We generated lentiviral sgRNA libraries and transfected them into hTSCs constitutively expressing Cas9. Using the resultant cells, we identified genes that regulate hTSC growth, EVT differentiation, and ST differentiation (Fig. 1*A* and Dataset S2). Two hTSC lines, CT27 and B31, were used for the screening. CT27 was derived from first-trimester placental tissue and B31 from a blastocyst (7). Similar results were obtained for the two independent cell lines (*SI Appendix*, Fig. S1*A*).

Our CRISPR screening identified 213 genes required for hTSC growth (Fig. 1*B*). These TFs include well-characterized genes essential for maintaining undifferentiated human trophoblasts, such as *GATA2/3*, *TFAP2C*, and *TEAD4* (15) (Dataset S2). We also identified eight growth-restricting genes including *CDKN1C* (Fig. 1*B*), which we previously reported as negatively regulating hTSC growth (16). Furthermore, our CRISPR screening identified 21 EVT-promoting and 54 ST-promoting genes, confirming *GCM1* as an EVT- and ST-promoting gene, *SNAI1* as an EVT-promoting gene, and *OVOL1* as an ST-promoting gene (17–20) (Fig. 1 *C* and *D*). We also identified 10 EVT-restricting and 16 ST-restricting genes.

To validate the reliability of our CRISPR screening, we compared our data with those of a recent study by Dong et al. (21), who conducted genome-wide CRISPR screening in hTSCs to identify growth-promoting and growth-restricting genes. It should be noted that Dong et al. did not analyze genes regulating hTSC differentiation. Focusing on the 850 genes analyzed in our study, we confirmed significant overlaps between the CRISPR screening results of the two studies (*P* = 3.1e-32 for growth-promoting genes, *P* = 9.7e-07 for growth-restricting genes; *SI Appendix*, Fig. S1*B* and Dataset S3). Another recent study by Chen et al. (8) identified 15 growth-promoting genes in hTSCs using RNAi screening. We found that the results by Chen et al. were not well aligned with those by us and Dong et al. (21) (*SI Appendix*, Fig. S1*C*). The cause of this discrepancy is unclear; however, Chen et al. and Dong et al. cultured hTSCs using the original hTSC medium developed by us (7). hTSCs cultured in the original medium showed enhanced toxicity following genetic manipulations such as lentiviral transfection or antibiotic selection (22). Therefore, in this study, we used the updated hTSC medium developed in our previous study to ameliorate the toxicity of genetic manipulations (22). Thus, we speculate that the side effects caused by genetic manipulations might partly explain the discrepancy observed in *SI Appendix*, Fig. S1*C*.

### Validation of DLX3 and GCM1 Function in hTSCs.

To further validate the results of our CRISPR screening, we decided to analyze two TFs, DLX3 and GCM1, for the following reasons. First, these were the only TFs required for both EVT and ST differentiation (Dataset S2), suggesting their critical role in human trophoblast differentiation. Second, although these TFs only regulate labyrinth development in mice (12, 13), they appeared to regulate both EVT and ST differentiation in humans. Third, the function of DLX3 in normal human trophoblasts is unclear. Finally, although recent studies have shown that GCM1 is required for EVT and ST differentiation (17, 18), the mechanism by which GCM1 regulates these processes remains elusive. Notably, the genome-wide binding sites of GCM1 have not been investigated in EVTs or STs.

We initially analyzed the expression patterns of DLX3 and GCM1 in human first-trimester placentas. DLX3 was expressed in the nuclei of all trophoblast cell types, whereas GCM1 was detected only in STs and EVTs (*SI Appendix*, Fig. S2*A*). We also confirmed that both *DLX3* and *GCM1* were highly expressed in EVTs differentiated from hTSCs (*SI Appendix*, Fig. S2*B* and Dataset S4). Meanwhile, their expression was transiently induced during the differentiation of hTSCs into STs. Although it is unclear whether *DLX3* and *GCM1* expression is also downregulated in mature STs in vivo, these data demonstrate trophoblast-specific expression of *DLX3* and *GCM1*, consistent with previous scRNA-Seq and single-nucleus RNA-Sequencing (snRNA-Seq) data (23, 24) (*SI Appendix*, Fig. S2 *C* and *D*).

**Fig. 2.**
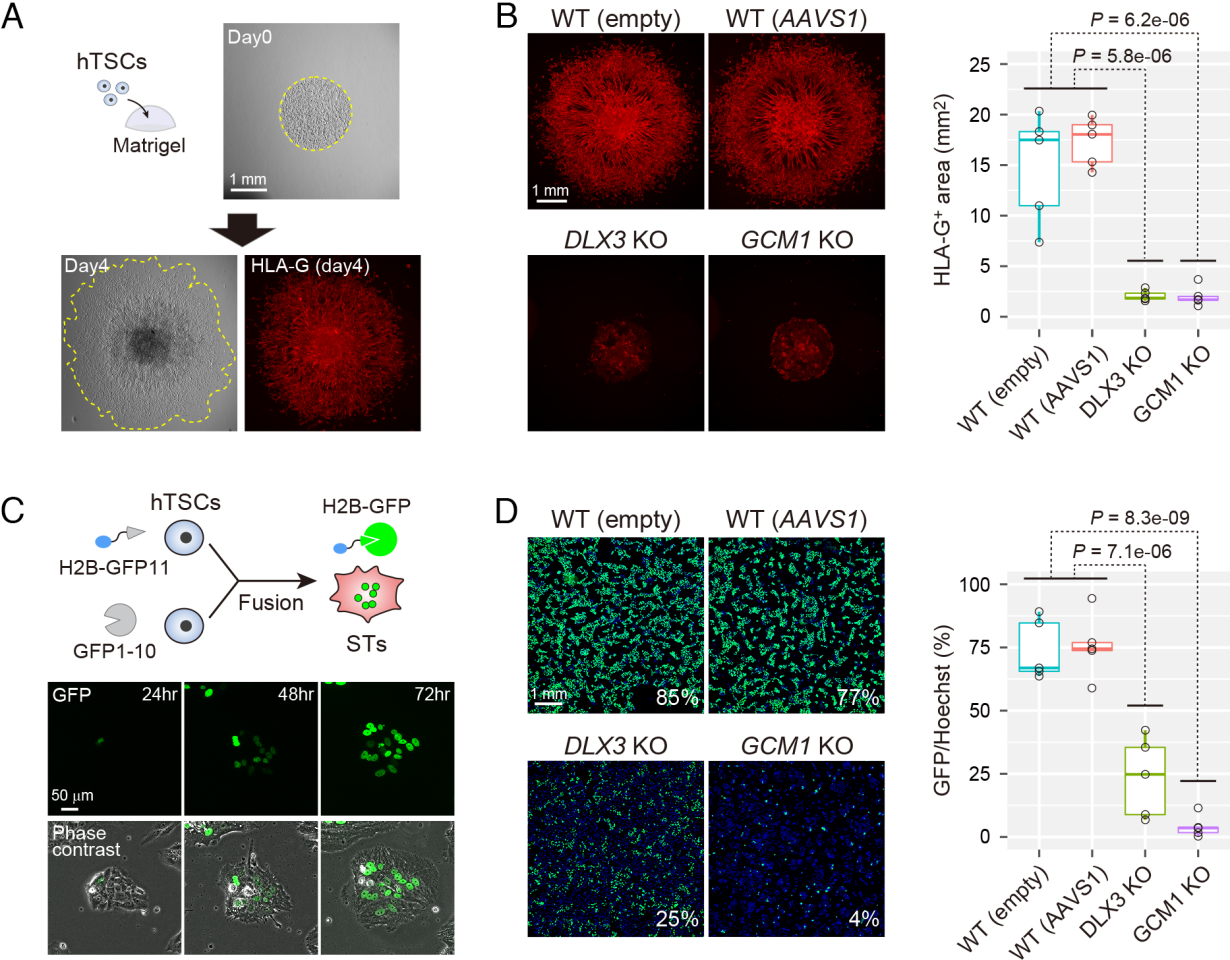
Functional analysis of *DLX3* and *GCM1* in hTSCs. (*A*) Quantification of EVT differentiation potential. Undifferentiated hTSCs were embedded in Matrigel drops and subjected to EVT differentiation. An anti-HLA-G antibody was used to detect EVTs. (*B*) EVT differentiation potentials of *DLX3* KO and *GCM1* KO clones. WT clones containing either an empty sgRNA vector or an AAVS1-targeting sgRNA vector were used as controls. We measured the HLA-G-positive area (red) to quantify the EVT differentiation potentials. Five independent clones were analyzed for each genotype. *P*-values were calculated using the Student’s *t* test. (*C*) Quantification of cell fusion efficiency. GFP11-labeled histone H2B was transfected into one pool of hTSCs, and GFP1-10 was transfected into another. These hTSC pools were mixed and subjected to ST differentiation. (*D*) ST differentiation potentials of *DLX3* KO and *GCM1* KO clones. We quantified the fusion efficiency by dividing the GFP-labeled area (green) by the Hoechst-labeled area (blue). Five independent clones were analyzed for each genotype. *P*-values were calculated using the Student’s *t* test.

To generate *DLX3* KO and *GCM1* KO hTSC clones, we employed CRISPR/Cas9 with sgRNAs designed to remove the DNA binding domains of DLX3 and GCM1 (*SI Appendix*, Fig. S2 *E* and *F*). After confirming the loss of DLX3 and GCM1 in the isolated KO clones, they were used for subsequent experiments. Wild-type (WT) hTSC clones containing either an empty sgRNA vector or an *AAVS1* (well-validated safe harbor locus)-targeting sgRNA vector were used as controls. To quantify the differentiation potential into EVTs, we developed a spread assay (Fig. 2*A*). We prepared drops of Matrigel containing undifferentiated hTSCs and cultured them in EVT differentiation medium. On day 4 after differentiation, we stained EVTs using an anti-HLA-G antibody and quantified the area occupied by HLA-G-positive cells. The results revealed that the WT clones efficiently differentiated into EVTs and migrated out of the Matrigel drops (Fig. 2*B*). In contrast, EVT differentiation and migration were severely compromised in both *DLX3* KO and *GCM1* KO clones.

We next analyzed whether *DLX3* KO and *GCM1* KO clones had the potential to differentiate into STs. To quantitatively assess ST differentiation, we used the split-GFP system (25). We transfected a lentivirus expressing GFP11-labeled histone H2B into one pool of hTSCs and transfected GFP1-10 into another pool. When these hTSC pools were mixed and subjected to ST differentiation, most nuclei were labeled with GFP (Fig. 2*C*). We quantified the fusion efficiency by dividing the GFP-labeled area by the Hoechst-labeled area. We found that both *DLX3* KO and *GCM1* KO clones had lower fusion efficiencies than those of WT clones, while the phenotype of *GCM1* KO clones was more severe than that of *DLX3* KO clones (Fig. 2*D*). Consistent with these results, STs differentiated from *DLX3* KO and *GCM1* KO clones showed decreased secretion of human chorionic gonadotropin (hCG), a hormone secreted by STs (*SI Appendix*, Fig. S2*G*). These results confirmed that DLX3 and GCM1 are essential for differentiation of hTSCs.

### Prediction of the Target Genes of DLX3 and GCM1.

**Fig. 3.**
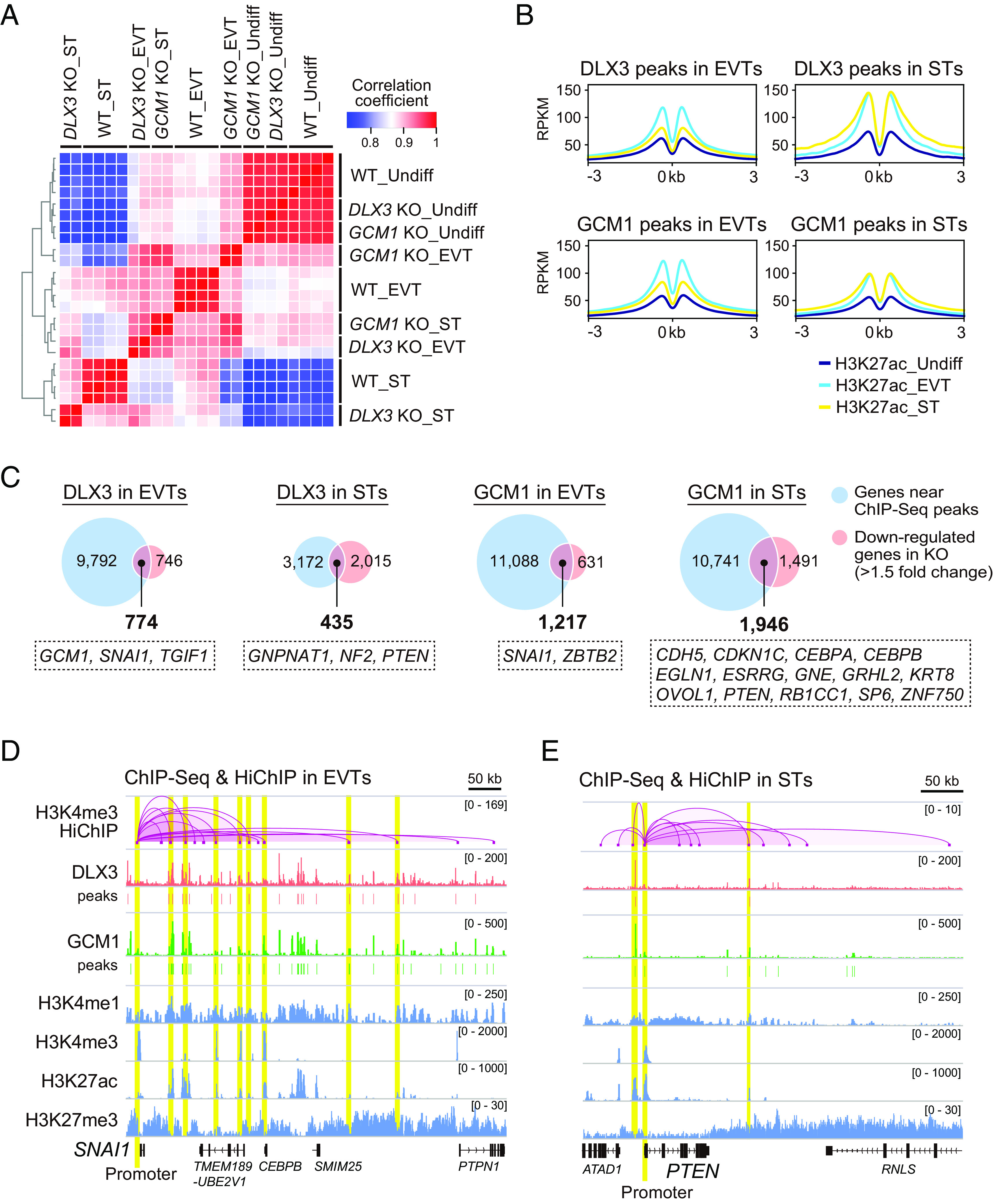
Prediction of DLX3 and GCM1 target genes. (*A*) Heatmap representation of Pearson correlation coefficients between WT, *DLX3* KO, and *GCM1* KO clones. We considered only genes with >5 TPM values in at least one of the analyzed cells. Two clones each of WT (empty), WT (AAVS1), *DLX3* KO, and *GCM1* KO lines were analyzed, including both undifferentiated (Undiff) and differentiated cells (EVTs or STs). (*B*) ChIP-Seq analysis of DLX3, GCM1, and H3K27ac. DLX3 and GCM1 binding sites were identified in both EVTs and STs. ChIP-Seq of H3K27ac was performed in undifferentiated hTSCs, EVTs, and STs. Averaged H3K27ac signals around DLX3 and GCM1 peaks are expressed as Reads Per Kilobase of exon per Million mapped reads (RPKM). Enriched motifs in the DLX3 and GCM1 ChIP-Seq peaks are indicated with their *P*-values and best-matched known TFs. (*C*) Prediction of DLX3 and GCM1 target genes in EVTs and STs. The genes near their ChIP-Seq peaks and those down-regulated by their respective KO were merged. Genes classified as EVT- or ST-promoting in our CRISPR screening are indicated in dotted boxes. (*D*) ChIP-Seq and HiChIP data in EVTs. The *SNAI1* locus is shown. The H3K4me3-marked *SNAI1* promoter is indicated in yellow. H3K27ac-marked enhancers that contained DLX3 and/or GCM1 peaks and physically interacted with the *SNAI1* promoter are also shown in yellow. The *y*-axis indicates RPKM for the ChIP-Seq data and −log_10_(q-value) for the HiChIP data. (*E*) ChIP-Seq and HiChIP data in STs. The *PTEN* locus is shown. Two enhancers containing DLX3 and GCM1 peaks physically interacted with the *PTEN* promoter (yellow).

To better understand how DLX3 and GCM1 regulate hTSC differentiation, we performed RNA-Seq of four WT, two *DLX3* KO, and two *GCM1* KO clones (Fig. 3*A* and Dataset S4). In the undifferentiated state, these clones exhibited similar gene expression profiles. However, as differentiation proceeded, their transcriptome profiles showed significant divergence. We analyzed differentially expressed genes (DEGs) between the WT and KO clones (*SI Appendix*, Fig. S3). This analysis revealed that EVT and ST markers were significantly down-regulated in differentiated KO clones. Instead, interferon response-related genes were aberrantly up-regulated in these KO clones, which could be caused by various cellular stresses (26).

We next performed ChIP-Seq of DLX3 and GCM1 in EVTs and STs derived from genetically unmodified hTSCs to determine their binding sites. After confirming strong correlations between biological replicates (*r* > 0.93), we identified the peaks shared between replicates (*SI Appendix*, Fig. S4 *A* and *B*). Motif analysis of the DLX3 peaks revealed that a previously reported DLX/HOX-binding motif was significantly enriched, whereas TEAD, GATA, and TFAP2 motifs were ranked higher than the DLX/HOX motif (*SI Appendix*, Fig. S4*C*). Similarly, analysis of the GCM1 peaks confirmed the significant enrichment of a previously reported GCM motif along with the TEAD, GATA, and TFAP2 motifs. Furthermore, we performed ChIP-Seq of four histone modifications in both undifferentiated and differentiated hTSCs: H3K4me1 (enhancer marker), H3K4me3 (promoter marker), H3K27ac (active enhancer marker), and H3K27me3 (repressive marker) (*SI Appendix*, Fig. S4*D*). We found that the DLX3 and GCM1 peaks in EVTs were associated with higher H3K27ac signals in EVTs than those in STs or undifferentiated hTSCs (Fig. 3*B*). DLX3 and GCM1 peaks in STs were associated with higher H3K27ac signals in STs and EVTs than those in undifferentiated hTSCs. In contrast, differentiation-dependent changes in signal intensities were less obvious for H3K4me1 and barely detectable for H3K4me3 and H3K27me3 (*SI Appendix*, Fig. S4*E*). Therefore, the binding of DLX3 and GCM1 is likely specifically linked to active enhancers, which is consistent with the transcriptional activation activities of DLX3 and GCM1 (27, 28).

To predict the target genes of DLX3 and GCM1, we merged the genes near their ChIP-Seq peaks with those down-regulated by their respective KO (Fig. 3*C* and Dataset S5). We identified 774 genes as potential targets of DLX3 in EVTs. Among these, *GCM1*, *SNAI1*, and *TGIF1* were classified as EVT-promoting genes in our CRISPR screening, suggesting that DLX3 may regulate EVT differentiation in part by activating *GCM1*, *SNAI1*, and *TGIF1*. Similarly, we identified 435, 1,217, and 1,946 genes as potential targets of DLX3 in STs, GCM1 in EVTs, and GCM1 in STs, respectively. Notably, some of these genes overlapped with EVT- or ST-promoting genes identified in our CRISPR screening (Fig. 3*C*). We also searched for pathways and cell types enriched among the potential target genes of DLX3 and GCM1 (*SI Appendix*, Fig. S5*A*). We found that EVT markers were enriched among the potential targets of DLX3 and GCM1 in EVTs, and ST markers were enriched among the potential targets of DLX3 and GCM1 in STs. We also compared the potential targets of DLX3 and GCM1 and found that the majority of potential targets of DLX3 were also targeted by GCM1 in both EVTs and STs (*SI Appendix*, Fig. S5*B*). Consistent with this finding, coimmunoprecipitation (Co-IP) experiments revealed a physical interaction between DLX3 and GCM1 in EVTs and STs (*SI Appendix*, Fig. S5*C*). These data suggested that DLX3 and GCM1 preferentially target genes that characterize EVTs and STs.

We then performed HiChIP for H3K4me3 to determine whether the binding sites of DLX3 and GCM1 physically interacted with the promoters of their potential target genes. We identified *SNAI1* as a potential target of both DLX3 and GCM1 in EVTs (Fig. 3*C* and *SI Appendix*, Fig. S6*A*). HiChIP analysis revealed interactions between the H3K4me3-labeled *SNAI1* promoter and several H3K27ac-labeled enhancers, some of which contained DLX3 and GCM1 binding peaks (Fig. 3*D*). We also examined the promoters of three well-characterized EVT regulators or markers (*GCM1*, *ASCL2*, and *HLA-G*) and confirmed that their promoters interacted with enhancers containing DLX3 and/or GCM1 peaks (*SI Appendix*, Fig. S6 *B*–*D*). HiChIP in STs also revealed interactions between the promoters of key ST regulators and enhancers containing DLX3 and GCM1 peaks. For example, we identified *PTEN* as a potential target of both DLX3 and GCM1 in STs (Fig. 3*C* and *SI Appendix*, Fig. S6*E*) and confirmed that the *PTEN* promoter interacted with two enhancers containing DLX3 and GCM1 peaks (Fig. 3*E*). We also confirmed that GCM1 binds the promoter region of *PGF* and regulates its expression (29) (*SI Appendix*, Fig. S6 *E* and *F*).

### Gene Function–Based Comparison of Human and Mouse Trophoblast Development.

**Fig. 4.**
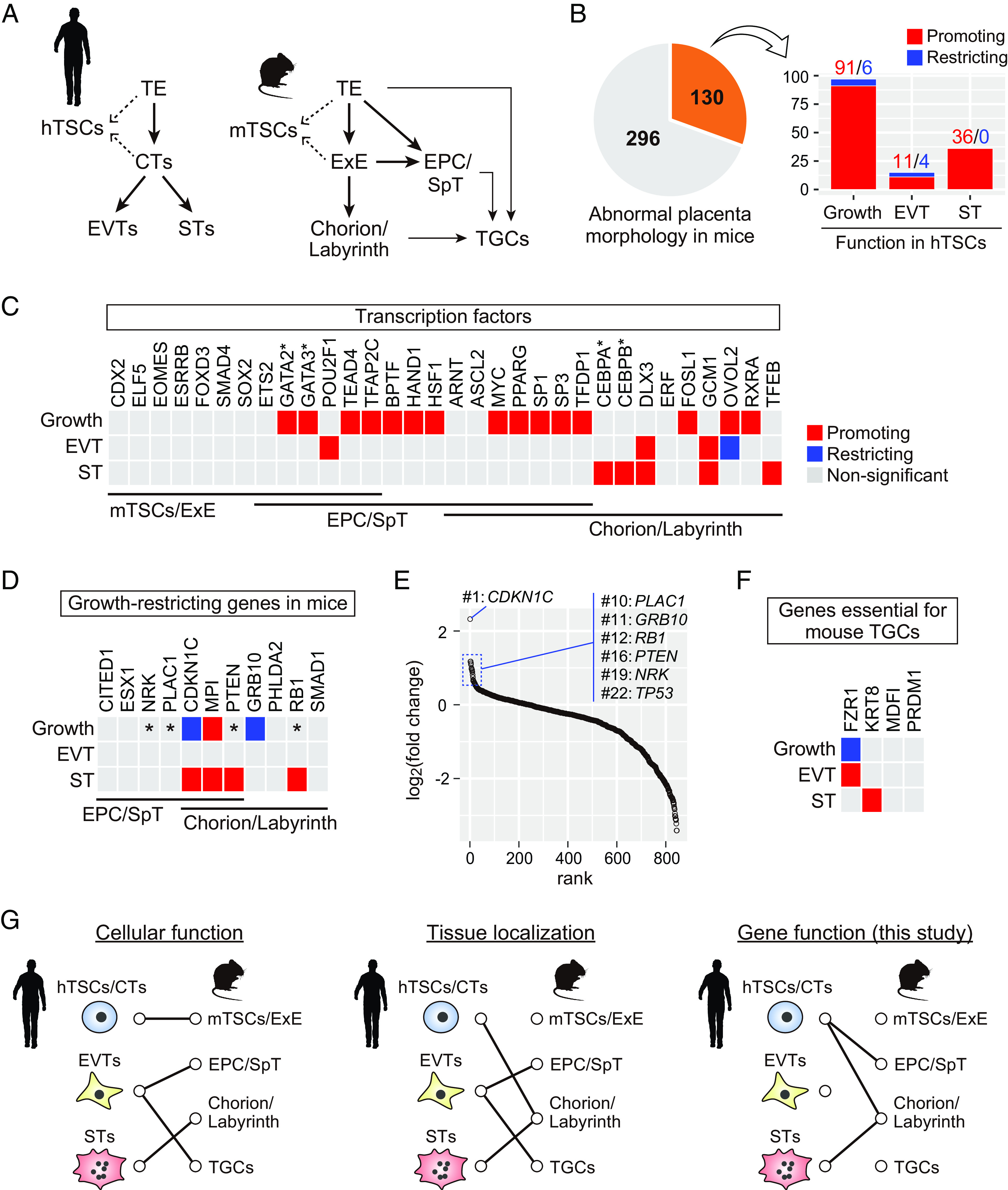
Comparison of gene functions between hTSCs and mouse placenta. (*A*) Schematic representation of human and mouse trophoblast development. (*B*) Identification of genes required in both hTSCs and mouse placenta. Among the 426 genes implicated in abnormal placenta morphology in mice, 130 genes were classified as essential for hTSC growth or differentiation. Genes that promote hTSC growth or differentiation are shown in red, and those that restrict hTSC growth or differentiation are in blue. (*C*) Analysis of TFs involved in the development of mTSCs/ExE, EPC/SpT, or chorion/labyrinth in mice. Genes promoting hTSC growth or differentiation are shown in red, genes restricting hTSC growth or differentiation are in blue, and those without significant effect are in gray. *Note that *Gata2/3* (30) and *Cebpa/b* (31) are functionally redundant in the mouse placenta. (*D*) Analysis of genes whose KO leads to an enlarged placenta in mice. Genes are color-coded as in (*C*). *Note that although not statistically significant (FDR > 0.05), these genes were highly ranked as negative growth regulators as shown in (*E*). (*E*) Results of CRISPR screening for hTSC growth regulators. Genes are ordered by their log_2_(fold change) values. Growth-restricting genes have high log_2_(fold change) values. (*F*) Analysis of genes essential for TGC differentiation in mice. Genes were color-coded as in (*C*). See also *SI Appendix*, Table S1 for a detailed listing of placental phenotypes of relevant KO mice. (*G*) (*Left*) Cellular function-based prediction. hTSCs/CTs share similarities with mTSC/ExE as both are proliferative and can give rise to differentiated trophoblasts. EVTs invade into the maternal uterus and remodel the spiral arteries. These functions are mediated by EPC/SpT and TGCs in mice. STs and the chorion/labyrinth mediate gas and nutrient exchange. (*Middle*) Tissue localization-based prediction. Human placental villi, which contain CTs and STs, are structurally similar to the chorion/labyrinth in mice. EVTs migrate out from placental villi and come into direct contact with uterine cells. Glycogen trophoblasts in the EPC/SpT and TGCs directly interact with uterine cells in the mouse placenta. (*Right*) Gene function–based prediction. Our CRISPR screening results suggest that hTSCs may be most similar to the EPC/SpT and chorion/labyrinth (possibly progenitor cells in these tissues), whereas STs are analogous to the chorion/labyrinth (possibly ST layer I and II). Alternatively, the exact counterpart of EVTs in mice could not be clearly determined.

To gain insights regarding the relationship between human and mouse trophoblast development, we compared the results obtained from our CRISPR screening with the phenotypes of previously reported mutant mice. In both humans and mice, all trophoblast cells originate from the TE (Fig. 4*A* and *SI Appendix*, Fig. S7). In humans, CTs arise from the TE following implantation and differentiate into EVTs and STs. In mice, the TE gives rise to the EPC, ExE, and trophoblast giant cells (TGCs) following implantation. The EPC and ExE-derived chorion contribute to the SpT and labyrinth layers, respectively. hTSCs are derived from blastocysts and CTs (7), whereas mTSCs are derived from blastocysts and the ExE (6). Our CRISPR screening classified 130 of the 426 genes implicated in abnormal placenta morphology in mice as essential for hTSC growth or differentiation (Fig. 4*B*). In subsequent analyses, we focused on genes that cause abnormalities in mTSCs and/or the ExE (mTSCs/ExE), the EPC and/or the SpT layer (EPC/SpT), or the chorion and/or the labyrinth layer (chorion/labyrinth), or TGCs upon KO in mice, and for which expression had been confirmed in affected tissues (see *SI Appendix*, Table S1 for details).

We first analyzed TFs and compared their KO phenotypes between hTSCs and mouse placenta (Fig. 4*C*). We found that none of the TFs specifically required for the maintenance of mTSCs/ExE (i.e., *CDX2*, *ELF5*, *EOMES*, *ESRRB*, *FOXD3*, *SMAD4*, and *SOX2*) were classified as significant in hTSCs. Among these, *CDX2*, *EOMES*, *ESRRB*, and *SOX2* were almost undetectable in hTSCs (<1 TPM) (Dataset S4). It should be noted that although *ETS2, GATA2/3*, *POU2F1*, *TEAD4*, and *TFAP2C* are also essential in mTSCs/ExE, these genes are expressed during and are essential for EPC/SpT development as well (30, 32–35). The results of our CRISPR screening suggested that almost all TFs required for EPC/SpT and/or chorion/labyrinth development were essential for hTSC growth or differentiation, excepting *ETS2*, *ERF*, *ARNT* (which had a relatively low FDR (0.10) in our CRISPR screening for growth regulators), and *ASCL2* (which had a relatively low FDR (0.15) in our CRISPR screening for EVT regulators) (Fig. 4*C* and Dataset S2). Most TFs required in the EPC/SpT and/or chorion/labyrinth were classified as growth-promoting in our CRISPR screening, implying that hTSC growth may require TFs that are essential for the growth of EPC/SpT and chorion/labyrinth. Additionally, more than half of the TFs (five of nine) specifically required for chorion/labyrinth development were identified as ST-promoting (Fig. 4*C*), suggesting a close link between ST differentiation and chorion/labyrinth development.

We next analyzed growth factor receptors and their downstream signal transducers (*SI Appendix*, Fig. S8). Although most of the analyzed genes were classified as nonsignificant in our CRISPR screening, we identified three significant genes: *EGFR*, *MAPK14*, and *FZD5*. *Egfr* KO mice have an extremely small EPC (36), and our CRISPR screening classified *EGFR* as growth-promoting, which is consistent with the essential role of EGF in the maintenance of hTSCs (7). In mice with KO of *Mapk14*, which encodes p38a, the labyrinth layer is almost completely lost, whereas the SpT layer is less affected (37, 38). CRISPR screening revealed that *MAPK14* functioned as a growth-restricting, EVT-restricting, and ST-promoting gene. *Fzd5* encodes a WNT receptor required for normal branching in the chorion (39). We identified *FZD5* as an ST-promoting gene. The functions of *MAPK14* and *FZD5* in hTSCs provide further evidence for a link between STs and the chorion/labyrinth.

Many genes cause placental enlargement when knocked out in mice; therefore, we investigated whether their KO had similar effects on hTSCs. Of the eleven genes whose KO in mice led to enlarged EPC/SpT and/or chorion/labyrinth, only two genes, *CDKN1C* and *GRB10*, were classified as growth-restricting in hTSCs by CRISPR screening (Fig. 4*D*). However, four other genes (i.e., *NRK*, *PLAC1*, *PTEN*, and *RB1*) were highly ranked as negative growth regulators in our CRISPR screening, although these did not reach statistical significance (Fig. 4*E*). These four genes had higher log_2_(fold change) values than *TP53*, which encodes the well-known tumor suppressor p53. Therefore, six of the eleven genes could function as growth-restricting genes in hTSCs. Additionally, among these six genes, three were also classified as ST-promoting genes (Fig. 4*E*). This may occur because ST differentiation is characterized by cell cycle exit (40) and defective ST differentiation may not counter enhanced hTSC growth. Overall, these data imply considerable overlap of negative growth regulators between hTSCs and the mouse placenta.

We finally analyzed genes essential for the development of TGCs. Because TGC defects are often secondary to abnormalities in the TE, ExE, or EPC/SpT, we focused only on genes that are not required for TE, ExE, and EPC/SpT development. Four genes, *FZR1*, *KRT8*, *MDFI*, and *PRDM1*, met our criteria (*SI Appendix*, Table S1). CRISPR screening identified *FZR1* and *KRT8* as EVT- and ST-promoting genes, respectively (Fig. 4*F*). Previous studies have shown that trophoblast cells in *Fzr1* KO mice do not undergo endoreplication, which refers to DNA replication without mitotic cell division and is essential for TGC differentiation (41, 42). Moreover, another study suggested that endoreplication may also be involved in EVT differentiation (43), which is consistent with our data.

## Discussion

In this study, we performed CRISPR screening to identify genes essential for hTSC growth and differentiation. Among them, we characterized *DLX3* and *GCM1* in detail and demonstrated their essential roles in hTSC differentiation. Previous studies utilizing choriocarcinoma or transformed cell lines have suggested that DLX3 and GCM1 physically interact and that DLX3 inhibits GCM1 transcriptional activation activity at the *PGF* locus (29, 44). We confirmed that DLX3 and GCM1 also interact in EVTs and STs (*SI Appendix*, Fig. S5*C*) and that GCM1 is required for the induction of *PGF* in both EVTs and STs (*SI Appendix*, Fig. S6*E*). However, we found that *PGF* expression remained unchanged in *DLX3* KO STs and was slightly down-regulated in *DLX3* KO EVTs (*SI Appendix*, Fig. S6*E*), which does not support the notion that DLX3 inhibits GCM1 transcriptional activation activity at the *PGF* locus. We also revealed that the majority of the potential target genes of DLX3 were also targeted by GCM1 and that both DLX3 and GCM1 binding were associated with increased H3K27ac signals (Fig. 3*B* and *SI Appendix*, Fig. S5*B*). Therefore, we propose that DLX3 and GCM1 work cooperatively rather than antagonistically to regulate EVT and ST differentiation. Moreover, recent studies using hTSCs have revealed that GCM1 is required for both EVT and ST differentiation and have identified a few target genes of GCM1, including *CKMT1* and *NOTUM* (17, 18). Our analyses not only confirm these previous findings (Dataset S4) but also provide a richer profile of GCM1 function.

A long-standing debate exists regarding the relationship between trophoblast development in humans and mice (3–5). Based on cellular function, hTSCs/CTs are similar to mTSCs/ExE (4–7) because they are proliferative and can give rise to differentiated trophoblasts (Fig. 4*G*). However, anatomically, CTs resemble progenitor cells in the chorion/labyrinth (3, 5) (Fig. 4*G*), because ExE-like structures are absent during human placental development. Previous studies suggested functional and structural similarities between EVTs and EPC derivatives (i.e., SpTs, glycogen trophoblasts, and TGCs). Several EVT subtypes exist in the human placenta, including cell column, interstitial, and endovascular subtypes. Cell column EVTs have been proposed to be similar to SpTs or parietal TGCs (3–5). Interstitial and endovascular EVTs might resemble glycogen trophoblasts and spiral artery TGCs in mice, respectively (3, 5). Human STs may be structurally and functionally similar to the ST layers I and II in the mouse labyrinth layer (3–5) (Fig. 4*G*).

Our comparative analysis classified all TFs specifically required for the maintenance of mTSCs/ExE as nonsignificant in hTSCs (Fig. 4*C*). Instead, many genes that positively or negatively regulate EPC/SpT and chorion/labyrinth development are required for normal hTSC growth (Fig. 4 *C* and *D*). Thus, hTSCs should have similarities with EPC/SpT and chorion/labyrinth cells. Notably, the EPC/SpT and chorion/labyrinth possess distinct progenitor cell populations. Previous studies have revealed that Blimp1-positive cells in the EPC exclusively contribute to glycogen trophoblasts and some TGC lineages (45), and that EPCAM-high cells in the chorion contribute only to labyrinth layer trophoblasts (46). Our data support the concept that hTSCs may be analogous to cells (possibly progenitor cells) in both the EPC/SpT and chorion/labyrinth (Fig. 4*G*). Consistent with this, previous studies have revealed that GATA2/3 and TEAD4 are essential for the maintenance of progenitor cells in the postimplantation mouse placenta (30, 34), and these TFs are also required for the maintenance of hTSCs (Fig. 4*C*). We also found that the ST-promoting genes are preferentially associated with chorion/labyrinth development (Fig. 4 *C* and *D*). Notably, although some ST-promoting genes are required for both EPC/SpT and chorion/labyrinth development, none are required exclusively for EPC/SpT development. Thus, these data support the analogy between STs and chorion/labyrinth cells (possibly ST layer I and II) (Fig. 4*G*).

Unlike hTSCs and STs, the analogous tissues of EVTs in mice could not be readily determined. Previous studies have demonstrated that *ASCL2* is required for EVT differentiation from hTSCs (47) and that *Ascl2* (also known as *Mash2*) is essential for SpT layer formation in mice (48), implying a potential link between EVTs and the SpT layer. However, with the exception of *POU2F1*, our CRISPR screening failed to identify additional genes that support a connection between EVT differentiation and EPC/SpT development. In addition, two EVT-promoting genes, *DLX3* and *GCM1*, are essential for chorion/labyrinth development, and an EVT-promoting gene, *Fzr1*, is required for TGC development in mice (41, 42). These data suggest that although EVTs may have some similarities to the EPC/SpT, chorion/labyrinth, and TGCs, it is less likely that a specific counterpart of EVTs exists in the mouse placenta.

Our gene function–based comparison of human and mouse trophoblast development has some limitations. First, although the results of CRISPR screening well reproduced previous findings, false positives and negatives are inevitable in this type of study. Second, a recent study suggested that EVTs derived from hTSCs may include cell column and interstitial EVTs but not endovascular EVTs and giant cells (23). Thus, our CRISPR screening was not suitable for identifying genes essential for endovascular EVT or giant cell differentiation. Finally, the EPC/SpT, chorion/labyrinth, and TGCs consist of multiple trophoblast subtypes; however, most previous studies on mutant mouse strains have not thoroughly characterized the proliferation and differentiation of each subtype. Therefore, it was difficult to analyze the relationship between human and mouse trophoblast subtypes in detail.

In conclusion, we compared the human and mouse trophoblast development based on gene function. We propose that hTSCs may be analogous to mouse EPC/SpT and chorion/labyrinth progenitor cells and that STs may be equivalent to mouse ST layer I and II in the chorion/labyrinth. These data will be useful in comparing human and mouse placental development. TSCs serve as an invaluable resource for analyzing trophoblast development, and we anticipate that conducting CRISPR screening in various mammalian TSCs will greatly advance our understanding of the molecular mechanisms underlying the evolution of mammalian placentas.

## Materials and Methods

First-trimester human placentas were obtained from healthy donors who provided written informed consent. All experimental protocols and procedures were approved by the Ethics Committee of Tohoku University Graduate School of Medicine (research license 2021-1-085). Two hTSC lines, CT27 and B31, were established in our previous study (16). CT27 was derived from CTs isolated from a first-trimester placenta, and B31 was derived from a blastocyst. Detailed materials and methods are included in *SI Appendix*.

## Supplementary Material

Appendix 01 (PDF)

Dataset S01 (XLSX)

Dataset S02 (XLSX)

Dataset S03 (XLSX)

Dataset S04 (XLSX)

Dataset S05 (XLSX)

Dataset S06 (XLSX)

Dataset S07 (XLSX)

## Data Availability

RNA-seq, ChIP-seq, and HiCHIP data have been deposited in Japanese Genotype–phenotype Archive (JGAS000107) (49) and National Center for Biotechnology Information (GSE244255) (50).
