# Supplementary material for: CRISPR screening in human trophoblast stem cells reveals both shared and distinct aspects of human and mouse placental development
Source: Proc Natl Acad Sci U S A. 2023 Dec 12;120(51):e2311372120. doi: 10.1073/pnas.2311372120 (PMC10742386; doi:10.1073/pnas.2311372120)
Supplement: Supplementary file 1 — Appendix 01 (PDF) [file pnas.2311372120.sapp.pdf]

PNAS

Supplemental text for

CRISPR screening in human trophoblast stem cells reveals both shared and distinct aspects of human and mouse placental development

This PDF file includes:

Supplemental Materials and Methods
Supplemental References
Supplemental Figures S1 to S8
Supplemental Table S1

Supplemental Materials and Methods

Culture of hTSCs

hTSCs were cultured as previously described (1). Briefly, hTSCs were maintained in hTSC medium [DMEM/F12 (FUJIFILM Wako #048-29785) supplemented with 1% knockout serum replacement (KSR) (Thermo Fisher Scientific #10828028), 0.5% penicillin-streptomycin (Thermo Fisher Scientific #15140122), 0.15% bovine serum albumin (BSA) (FUJIFILM Wako #017-22231), 1% ITS-X supplement (FUJIFILM Wako #094-06761), 200 µM L-ascorbic acid (FUJIFILM Wako #013-12061), 25 ng/ml EGF (FUJIFILM Wako #053-07871), 2 µM CHIR99021 (FUJIFILM Wako #038-23101), 5 µM A83-01 (FUJIFILM Wako #035-24113), 0.8 mM valproic acid (FUJIFILM Wako #227-01071), and 2.5 µM Y27632 (FUJIFILM Wako #257-00511)] at 37°C in 95% air and 5% CO_2. We reduced the concentration of EGF from 50 ng/ml (1) to 25 ng/ml because the lower concentration is sufficient to maintain hTSCs and thus cost-effective. When hTSCs reached sub-confluence, they were dissociated with TrypLE Express (Thermo Fisher Scientific #12604021) diluted with PBS at a 1:1 ratio for 10-15 min at 37°C. Dissociated cells were seeded in hTSC medium supplemented with 0.5 µg/ml iMatrix-511 (Nippi #892011). hTSCs were typically passaged every two days at a split ratio of 1:4 to 1:8. For CRISPR screening and gene KO, 10 ng/ml BMP4 (R&D Systems #314-BP) was added to the hTSC medium to ameliorate the toxicity of gene transfection and antibiotic selection.

sgRNA library construction

The kanamycin/neomycin-resistance gene was PCR amplified from pCAG-HIVgp (RIKEN BioResource Center, kindly provided by Dr. H. Miyoshi) using KAPA HiFi HS ReadyMix (KAPA #KK2601) and cloned downstream of the sgRNA scaffold sequence of pCS-hU6 (1) to generate pCS-hU6-Neo. The sgRNA sequences were obtained from a previous study (2), and their gene-specific regions (TGG AAA GGA CGA AAC ACC G N_{20} GTT TCA GAG CTA TGC TGG AAA, where N_{20} is the target sequence) were chemically synthesized (GenScript). These oligos were PCR amplified and cloned into Pst1-digested pCS-hU6-Neo using HiFi DNA Assembly Master Mix (NEB

#E2621). The resultant sgRNA library was amplified using NEB 5-alpha Competent E. coli (NEB #C2987). The oligo sequences used for library construction are shown in Dataset S6.

To generate lentivirus expressing a pooled sgRNA library, the library was co-transfected with pCMV-VSV-G-RSV-Rev and pCAG-HIVgp (RIKEN BioResource Center, kindly provided by Dr. H. Miyoshi) into 293T cells using CalFectin (SignaGen #SL100478). At 24 h following transfection, 10 µM forskolin (FUJIFILM Wako #067-02191) was added. The supernatant was collected after three days of transfection, concentrated using Lenti-X Concentrator (Takara #631231), and stored at -80°C.

We constructed two sgRNA libraries, one targeting TFs and the other targeting genes essential for mouse placental development. These genes were selected as follows: a list of human TFs was obtained from a previous study (3), of which 458 TFs had intermediate or high expression levels in primary human trophoblasts or hTSCs [>20 TPM in at least one cell type based on our previous study (4)]. To identify the human orthologs of genes essential for mouse placental development, we searched the MGI database (http://www.informatics.jax.org) for genes that cause "abnormal placenta morphology" when mutated in mice. We excluded mice that had mutations in two or more genes or exhibited phenotypes only in non-trophoblast cells such as decidua and fetal blood cells. In total, 426 genes were identified. Of these, 34 were already included among the 458 TFs already identified; the remaining 392 genes, including 20 TFs, were incorporated into the library targeting genes essential for mouse placental development. Overall, the library targeting TFs contained 1882 sgRNAs, including 50 non-targeting sgRNAs. The other library contained 1668 sgRNAs, including 100 control sgRNAs targeting genes that were unexpressed (<1 TPM) in primary human trophoblasts and hTSCs (Dataset S1).

CRISPR screening in hTSCs

The puromycin-resistance gene (PuroR) and Cas9 were PCR amplified from pGIPZ (Open Biosystems) and the Alt-R S.p. Cas9 Expression Plasmid (IDT), respectively. PuroR, a T2A element, and Cas9 were cloned into the CS-CA-MCS plasmid (RIKEN BioResource Center, kindly provided by Dr. H. Miyoshi) using In-Fusion HD Cloning Kit (Takara # 639648). The resulting vector was named pCS-CA-PuroR-T2A-Cas9. A lentivirus expressing PuroR-T2A-Cas9 was prepared as described above and transduced into the two hTSC lines, CT27 and B31. Cas9-expressing hTSCs were selected with 2 µg/ml puromycin (Thermo Fisher Scientific #A1113803). Lentivirus expressing a pooled sgRNA library was transfected into the Cas9-expressing hTSCs. For each sgRNA library, three million Cas9-expressing hTSCs were used for transfection, which was sufficient to achieve coverage of 500 cells per sgRNA. At 24 h after transfection, half of the cells were collected for genomic DNA isolation. The other half were passaged and selected with 400 µg/ml G418 (Sigma #G8168 or Nacalai #09380-86) for five days to concentrate sgRNA-expressing cells. To avoid the expression of multiple sgRNAs in a single cell, the lentivirus concentration was adjusted to kill approximately two-thirds of the transfected cells during G418 selection.

To identify hTSC growth regulators, sgRNA-transfected cells were maintained for 20 days and collected for genomic DNA isolation. This time period was determined based on a previous CRISPR screening study using hTSCs (5). To identify regulators of EVT differentiation, sgRNA-transfected cells were maintained for 15 days in hTSC medium and then differentiated into EVTs as previously reported (4) with minor modifications. Briefly, hTSCs were cultured in EVT medium [DMEM/F12 supplemented with 0.5% penicillin-streptomycin, 0.15% BSA, 1% ITS-X supplement, 50 ng/ml NRG1 (Cell Signaling #5218SC or #26941), 7.5 µM A83-01, 2.5 µM Y-27632, and 4% KSR] supplemented with 2% Matrigel (Corning #354263) for three days. The culture medium was then replaced with EVT medium without NRG1. EVTs were collected on day 5 using a phycoerythrin (PE)-labeled anti-HLA-G antibody (Abcam clone #MEM-G/9) and EasySep™ Human PE Positive Selection Kit (Veritas #ST-18551) for genomic DNA isolation. To identify regulators of ST differentiation, sgRNA-transfected cells were maintained for 15 days in hTSC medium and then differentiated into STs as previously reported (4) with minor modifications. Briefly,

hTSCs were cultured on plates coated with 1 µg/ml Col IV (Corning #354233) using ST medium [DMEM/F12 supplemented with 0.5% penicillin-streptomycin, 0.15% BSA, 1% ITS-X supplement, 2.5 µM Y-27632, 2 µM forskolin, and 4% KSR] for two days. STs (>40 µm) were then separated from unfused or poorly fused (<40 µm) cells using a 40 µm cell strainer (Corning #352340). Cells of both sizes were collected for genomic DNA isolation. The maintenance and differentiation of hTSCs were performed at 37°C in 95% air and 5% CO_2.

Genomic DNA was isolated from the collected cells using AllPrep DNA/RNA Mini Kit (Qiagen #80204). Target sequences of the sgRNAs were PCR-amplified using KOD FX Neo (TOYOBO #KFX-201) and purified using DNA Clean & Concentrator Kit (Zymo Research #D4013). The PCR products were indexed with Dual Index primers (IDT # 10009816) and subjected to sequencing on an Illumina NovaSeq 6000 platform (Illumina) with 150 bp paired-end reads (Rhelixa). The obtained reads were aligned to the sgRNA target sequences and analyzed using MAGeCK v0.5.9.5 (6). Data obtained from the CT27 and B31 cells were treated as biological replicates. The Gini indices were sufficiently low (<0.07), indicating the evenness of the sgRNA read counts (Dataset S1). The oligo sequences used to amplify the sgRNA target sequences are shown in Dataset S6.

The read counts of the 50 non-targeting sgRNAs contained within the TF-targeting sgRNA library were significantly increased in day 20 hTSCs compared to those in day 1 hTSCs (Dataset S1). This phenomenon may be explained by the toxicity of Cas9-induced double-strand breaks because non-targeting sgRNAs may not afford such toxicity (7). Therefore, to identify hTSC growth regulators, genes that were not expressed (<1 TPM) in hTSCs were used as controls (20 sgRNAs targeting *DLX6*, *KLF2*, *NR4A3*, *STAT5A*, and *ZNF439*). To identify regulators of EVT and ST differentiation, the non-targeting sgRNAs were used as controls because their read counts were comparable among day 20 hTSCs, EVTs, and STs (Dataset S1). In turn, the mouse placental development gene-targeting sgRNA library included 100 control sgRNAs targeting genes that were not expressed (<1 TPM) in primary human trophoblasts and hTSCs. These control sgRNAs were used to identify genes essential for hTSC growth and differentiation.

Immunohistochemistry

Human placental biopsies obtained from donors at 6-9 weeks of gestation were fixed overnight at 4°C in 4% paraformaldehyde (PFA). The fixed biopsies were sectioned using a Leica MURUTICUT R microtome (Leica) to a thickness of 5 µm. Antigen retrieval was performed at 90°C for 20 min using HistoVT One (Nacalai Tesque #06380), and nonspecific signals were blocked with PBS-T (PBS with 1% Tween 20) containing 2% BSA (FUJIFILM Wako #017-22231). Anti-DLX3 (Abcam #ab178428) and anti-GCM1 (Sigma #HPA011343) antibodies were diluted to 1:1000 and 1:250 in PBS-T, respectively. A horseradish peroxidase (HRP)-labeled polymer conjugated secondary antibody (MBL #8460) was placed directly on the sections, and 3,3'-diaminobenzidine (DAB) Substrate Solution (MBL #8469) was used as the substrate for HRP. Sections were imaged using a fluorescence microscope (BZ-X800, Keyence).

Generation of *DLX3* KO and *GCM1* KO clones

To generate *DLX3* KO and *GCM1* KO hTSCs, sgRNAs were cloned into pCS-hU6-Neo as follows. The sgRNA scaffold sequence and mouse U6 promoter (sgRNA-mU6) were synthesized using a custom gene synthesis service (Eurofins Genomics). Two sgRNA target sequences flanking an exon of *DLX3* or *GCM1* (Figs. S2E and F) were added to sgRNA-mU6 using PCR and cloned into pCS-hU6-Neo. The resulting vector was co-transfected into 293T cells with pCMV-VSV-G-RSV-Rev and pCAG-HIVgp to generate lentivirus. The lentivirus was transduced into Cas9-expressing CT27 hTSCs, and sgRNA-expressing cells were selected using G418. Following single-cell cloning, *DLX3* KO and *GCM1* KO hTSC clones were identified using PCR. The oligo sequences used to generate the KO hTSCs are shown in Dataset S6.

Western blotting

hTSCs were differentiated into EVTs as described above, lysed in Sample Buffer Solution (Wako #196-11022), homogenized using an ultrasonic reactor (Sonics&Materials #VCX-130), and denatured at 95°C for 10 min. The cell lysates were electrophoresed on a Mini-PROTEAN TGX gel (Bio-Rad #4561633). Precision Plus Protein All Blue Standards (BIO-RAD #1610373) were used as molecular standards. The electrophoresed proteins were transferred to a polyvinylidene-fluoride (PVDF) membrane (Cytiva #10600038) and blocked with a blocking solution [TBS (pH 7.4) with 5% skim milk and 0.05% Tween 20] at room temperature (approximately 24 °C) for 60 min. After blocking, the membrane was incubated in Can Get Signal Solution 1 (TOYOBO #29018903) containing primary antibodies overnight at 4°C. The following primary antibodies were used: anti-DLX3 (Abcam #ab178428, diluted 1:1000), anti-GCM1 (Sigma #HPA011343, diluted 1:200), and anti-β-Actin (Cell Signaling Technology #4967, diluted 1:1000). After washing thrice with TBS-T (TBS with 0.05% Tween 20), the membrane was incubated in Can Get Signal Solution 2 (TOYOBO #29018904) containing HRP-linked anti-rabbit IgG (GE Healthcare #NA934, diluted at 1:1000) at room temperature for 60 min. After washing thrice with TBS-T, chemiluminescence was detected using ECL Prime Western Blotting Detection Reagent (Cytiva #RPN2236) with a ChemiDoc MP (Bio-Rad).

Enzyme-linked immunosorbent assay (ELISA)

hTSCs were differentiated into STs for four days using ST medium. The supernatants were collected and the amount of secreted hCG was measured using hCG ELISA kit (Abnova #KA4005). The absorbance was measured using an AMR-100 microplate reader (Hangzhou Allsheng Instruments Co.,Ltd.).

RNA-Seq

Genetically unmodified hTSCs were differentiated into EVTs for five days and STs for four days, as described above. WT, *DLX3* KO, and *GCM1* KO clones were differentiated into EVTs for three days and STs for two days. Defects in the differentiation of these KO clones were evident at these time points. Total RNA was extracted using RNeasy Mini Kit and RNase-Free DNase (QIAGEN #74536 and #79254). RNA integrity (RINe) values were measured using a TapeStation 2200 (Agilent Technologies), and all samples were confirmed to have RINe values of >9. RNA-Seq libraries were constructed using NEBNext UltraII Directional RNA Library Prep Kit (NEB) and sequenced on an Illumina NovaSeq 6000 platform with 150 bp paired-end reads (Rhelixa). Sequenced reads were trimmed for quality control using TrimGalore v0.6.7 and aligned to the reference genome (UCSC hg38) using STAR v2.7.10a (8) with the RefSeq gene annotation. The expression levels (TPM) of RefSeq genes were calculated using RSEM v1.3.1 (9). Read counts were used to identify DEGs with the software DESeq2 v1.36.0 (10). PCA was performed using the prcomp function in R v4.2.2 (http://www.R-project.org/). Transcripts less than 300 bp in length (encoding microRNAs or small nucleolar RNAs in most cases) were excluded from the analysis.

Quantitative analysis of EVT differentiation

To quantify EVT differentiation, 10 µl of Matrigel and 10 µl of basal medium [DMEM/F12 supplemented with 0.5% penicillin-streptomycin, 0.15% BSA, 1% ITS-X supplement, and 1% KSR] containing 150,000 undifferentiated hTSCs were mixed. Then, 1 µl of the mixture was gelled on a 35 mm dish (IWAKI #3000-035) to form a Matrigel drop; we prepared four or five Matrigel drops for each hTSC clone. These drops were cultured in EVT medium supplemented with 0.5% Matrigel at 37°C in 95% air and 5% CO_2 for two days. The medium was then replaced with the basal medium supplemented with 0.5% Matrigel, and the culture was continued at 37°C in 5% O_2 and 5% CO_2 for another two days. The hypoxic condition enhanced EVT differentiation and invasion from Matrigel drops. On day 4, a PE-labeled anti-HLA-G antibody (Abcam #ab24384) was added directly to the medium at 1:300 dilution; the culture was further incubated at 37°C for 15 min, then fixed with 4%

PFA. After washing with PBS, the cells were examined using a BZ-X710 fluorescence microscope (Keyence), and the PE-labeled area was quantified using a BZ-X analyzer (Keyence). For each hTSC clone, four or five Matrigel drops were analyzed, and their data were averaged.

Quantitative analysis of ST differentiation

A split GFP system (51) was used to quantify the efficiency of cell fusion. GFP11-labeled histone *H2B* and *GFP1-10* were synthesized using a custom gene synthesis service (Eurofins Genomics) and cloned into the CS-CA-MCS plasmid to generate pCS-CA-GFP11-H2B and pCS-CA-GFP1-10, respectively. Lentivirus expressing GFP11-H2B or GFP1-10 was generated as described previously. Then, lentivirus expressing GFP11-H2B was transduced into one pool of hTSCs, and a lentivirus expressing GFP1-10 into another pool. The hTSC pools were mixed and cultured in the ST medium at 37°C in 95% air and 5% CO_2 for three days. Differentiated cells were fixed with 4% PFA for 15 min, counterstained with Hoechst 33258 (DOJINDO #H341), and analyzed using the BZ-X710 fluorescence microscope. The GFP- and Hoechst-labeled areas were measured using the BZ-X analyzer. To quantify cell fusion efficiency, the GFP-labeled area was divided by the Hoechst-labeled area. The oligo sequences used for vector construction are shown in Dataset S6.

ChIP-Seq

ChIP was performed using ChIP Reagents (NIPPON GENE #318-07131) and antibodies against DLX3 (Abcam #ab178428, diluted 1:100), GCM1 (Sigma #HPA011343, diluted 1:100), H3K4me1 (Cell Signaling Technology, Clone #D1A9, diluted 1:500), H3K27ac (MBL, Clone #CMA309, diluted 1:150), and H3K27me3 (MBL, Clone #CMA323, diluted 1:500). For ChIP of DLX3, H3K4me1, H3K27ac, and H3K27me3, IP was performed in an IP buffer containing 400 mM NaCl instead of 150 mM to reduce the background signal. ChIP-Seq libraries of histone modifications were constructed and sequenced on the Illumina HiSeq 2500 platform with 101-bp paired-end reads as previously described (11). ChIP-Seq libraries of TFs were constructed using NEBNext Ultra II DNA Library Prep Kit for Illumina (NEB # E7645) with two custom adaptors: Adapter-T and Adapter-C (12). Adapter-T with a 3′-T overhang was prepared by annealing (5′-CTA CAC GAC GCT CTT CCG ATC TT-3′) and (5′-AGA TCG GAA GAG CAC ACG TCT GAA-3′; 5′ phosphorylated). Adapter-C with a 3′-C overhang was prepared by annealing (5′- CTA CAC GAC GCT CTT CCG ATC TC-3′) and (5′-AGA TCG GAA GAG CAC ACG TCT GAA-3′; 5′ phosphorylated). Adapter-C was added to increase the ligation efficiency. The ChIP-Seq libraries of the TFs were sequenced on the Illumina Hiseq2500 or NovaSeq 6000 platform with single or paired-end reads. ChIP-Seq reads (only forward reads were used in this study) were trimmed for quality control using TrimGalore v0.6.7 and mapped to the reference genome (UCSC hg38) using Bowtie 2 v2.2.5 (13). TF-binding sites were identified using MACS2 v.2.2.7.1 (14), and de novo motifs within peaks were identified using HOMER (15). The ENCODE blacklist (16), a set of regions that exhibit anomalous, unstructured, or high signal in next-generation sequencing experiments independent of cell lines or experiments, was excluded from the analysis.

Co-immunoprecipitation (Co-IP)

hTSCs were differentiated into STs for two days or EVTs for five days as described above. Cells were washed three times with PBS and lysed in PierceTM IP Lysis Buffer (Thermo Scientific #87787) containing cOmplete protein inhibitor cocktail (Roche #11836153001). A total of 2.5~3.0 mg of protein was subjected to IP. Four micrograms of anti-DLX3 antibody (Abcam #ab178428) and normal rabbit IgG (FUJIFILM Wako #148-09551) were coupled with Dynabeads M280 sheep anti-Rabbit (Invitrogen #11203D) overnight at 4 ºC. IP was performed at 4°C for 4h, and IP products were separated from the magnetic beads by denaturation at 95 ºC for 10 min. Western blotting was performed using an anti-GCM1 antibody (Sigma #HPA011343) and VeriBlot for IP Detection Regent (Abcam #ab131366). Chemiluminescence was detected using ECL Prime Western Blotting Detection Reagent (Cytiva #RPN2236) with a FUSION SL (Vilber Lourmat).

HiChIP

HiChIP libraries were generated as previously reported (17-19) with minor modifications. Briefly, approximately 10 million cells were fixed in 1% PFA for 10 min and 3 mM disuccinimidyl glutarate for 40 min as previously described. Fixed NIH3T3 cells were added to the cross-linked cells as spike-in controls. The cell mixtures were lysed in HiC Lysis Buffer [10 mM Tris-HCl (pH 7.5), 10 mM NaCl, and 0.2% IGEPAL CA-630], and the isolated nuclei were resuspended in 0.5% sodium dodecyl sulfate (SDS) and permeabilized at 62°C for 10 min. Next, a 3.3-fold volume of 1.5% Triton X-100 was added and incubated at 37°C for 15 min. In situ digestion with DpnII (NEB #R0543) was performed at 37°C for 60 min, and the enzyme was heat-inactivated at 62°C for 20 min. Overhangs were biotin-labeled using DNA Polymerase I large (Klenow) Fragment (NEB #M0210) at 37°C for 60 min in the presence of biotin-14-dATP (Thermo Fisher Scientific #19524016), dTTP, dCTP, and dGTP. Proximity ends were ligated with 13.3 units/µl of T4 DNA ligase (NEB #M0202) at 23°C for 4 h. The nuclei were pelleted and treated with 0.5 units/µL of Exonuclease III (NEB #M0206S) for 5 min at 37°C, resuspended in Nuclear Lysis Buffer [50 mM Tris (pH7.5), 10 mM EDTA, and 1% SDS], and sonicated using Covaris M220 (M&S Instruments Inc.) to 300-700 bp length. Sonicated DNA was diluted 1:10 with ChIP Dilution Buffer [50 mM Tris (pH 7.5), 165 mM NaCl, 1.1% Triton X-100, and 0.01% SDS] and clarified by centrifugation. An anti-H3K4me3 antibody (Merck Millipore, Clone #CMA304) was added to the fragmented DNA at 1:500 dilution, and the mixture was rotated at 4 °C for 60 min. The IP complex was captured using Dynabeads M280 anti-mouse (Life technologies #11201D), and the beads were washed four times with High-Salt Wash Buffer [20 mM Tris (pH 7.5), 500 mM NaCl, 2 mM EDTA, 1% Triton X-100, 0.1% SDS, and 0.1% sodium deoxycholate] and twice with 10 mM Tris-HCl (pH7.5). The washed beads were suspended in Extraction Buffer [10 mM Tris (pH8.0), 350 mM NaCl, 0.1 mM EDTA, and 1% SDS] supplemented with 5% volume of proteinase K (Takara, #9034), and incubated at 55°C for 1 h and 67°C for 2 h to reverse the crosslinks. ChIPed DNA was purified using MinElute PCR Purification Kit (QIAGEN #28004). For biotin pull-down, 5 µl of Dynabeads MyOne Streptavidin C1 beads (Life Technologies #65001) were suspended in 2x Biotin Binding Buffer [10 mM Tris-HCl (pH 7.5), 1 mM EDTA, and 2 M NaCl] and mixed with an equal volume of ChIPed DNA. After 50 min of rotation at room temperature, the beads were washed with Tween Wash Buffer [5 mM Tris-HCl (pH 7.5), 0.5 mM EDTA, 1 M NaCl, 0.05% Tween-20] and NEB T4 Ligase Buffer (NEB # B0202), and resuspended in 10 mM Tris-HCl (pH 8.0). HiChIP libraries were constructed using KAPA Hyper Prep Kit (NIPPON Genetics #7962312001) and TruSeq-compatible duplex Y adapter (IDT). After PCR amplification, the amplicon was size-selected using Ampure XP (Beckman Coulter #A63880) and sequenced on the Illumina NovaSeq 6000 platform with 150 bp paired-end reads (Rhelixa). The amounts of DNA used for HiChIP are summarized in Dataset S7.

HiChIP reads were mapped to the reference genome (UCSC hg38) using the HiC-Pro pipeline (20) with default settings. All valid pairs obtained from the replicates were merged, and reproducibility was evaluated using HiCRep (21). The chromatin interactions were highly reproducible between the merged data and each replicate (stratum adjusted correlation coefficient > 0.8 when the smoothing parameter h was 3 and the maximum distance was 1000 kb). Therefore, only merged data were used for the analysis in the main text. Significant long-range (>20 kb) chromatin interactions were calculated using FitHiChIP (22). H3K4me3 ChIP-Seq data from hTSCs, hTSC-derived STs, and hTSC-derived EVTs were used as inputs for FitHiChIP. Only interactions with at least one end overlapping the H3K4me3 ChIP-Seq peaks were retained. After merged filtering, BEDPE files were used for visualization.

External data

The RNA-Seq data used to select genes for CRISPR screening were obtained from our previous study (4) and were processed as described above. The ChIP-Seq data for H3K4me3 were obtained from our previous study (11). The scRNA-Seq and snRNA-Seq data for first-trimester human placentas were obtained from https://www.reproductivecellatlas.org (23, 24).

Graphical presentation

ChIP-Seq and HiChIP data were visualized using deeptools v3.5.1 (25) and Integrative Genomics Viewer (IGV) v2.8.0 (26). Bar charts, scatterplots, boxplots, volcano plots, and heatmaps were generated using the ggplot2 and pvclust packages in R.

Statistical analysis

The statistical analyses of CRISPR screening results, DEGs, ChIP-seq peaks, sequence motifs, enriched pathways and cell types, and HiChIP interactions were performed using MAGeCK (6), DESeq2 (10), MACS2 (14), HOMER (15), Enrichr (27), and FitHiChIP (22), respectively. The statistical analyses in Fig. S1B and Figs. 2B, 2D, and S2G were performed using Fisher's exact test and Student's t-test, respectively. A *P*- or q-value < 0.05 was considered statistically significant.

Data and materials availability

Histone ChIP-Seq data are deposited in the Japanese Genotype-phenotype Archive (JGA) under the accession number JGAS000107 [https://humandbs.biosciencedbc.jp/en/hum0086-v3]. RNA-seq, TF ChIP-Seq, and HiChIP data are deposited in NCBI under the accession number GSE244255 [https://www.ncbi.nlm.nih.gov/geo/query/acc.cgi?acc=GSE244255]. Other data needed to evaluate the conclusions in this paper are preset in the paper and/or the Supplementary Materials.

References

1. S. Takahashi *et al.*, Loss of p57(KIP2) expression confers resistance to contact inhibition in human androgenetic trophoblast stem cells. *Proc Natl Acad Sci U S A* **116**, 26606-26613 (2019).
2. J. G. Doench *et al.*, Optimized sgRNA design to maximize activity and minimize off-target effects of CRISPR-Cas9. *Nat Biotechnol* **34**, 184-191 (2016).
3. S. A. Lambert *et al.*, The Human Transcription Factors. *Cell* **172**, 650-665 (2018).
4. H. Okae *et al.*, Derivation of Human Trophoblast Stem Cells. *Cell Stem Cell* **22**, 50-63 e56 (2018).
5. C. Dong *et al.*, A genome-wide CRISPR-Cas9 knockout screen identifies essential and growth-restricting genes in human trophoblast stem cells. *Nat Commun* **13**, 2548 (2022).
6. W. Li *et al.*, MAGeCK enables robust identification of essential genes from genome-scale CRISPR/Cas9 knockout screens. *Genome Biol* **15**, 554 (2014).
7. D. W. Morgens *et al.*, Genome-scale measurement of off-target activity using Cas9 toxicity in high-throughput screens. *Nat Commun* **8**, 15178 (2017).
8. A. Dobin *et al.*, STAR: ultrafast universal RNA-seq aligner. *Bioinformatics* **29**, 15-21 (2013).
9. B. Li, C. N. Dewey, RSEM: accurate transcript quantification from RNA-Seq data with or without a reference genome. *BMC Bioinformatics* **12**, 323 (2011).
10. M. I. Love, W. Huber, S. Anders, Moderated estimation of fold change and dispersion for RNA-seq data with DESeq2. *Genome Biol* **15**, 550 (2014).
11. N. Kobayashi *et al.*, The microRNA cluster C19MC confers differentiation potential into trophoblast lineages upon human pluripotent stem cells. *Nat Commun* **13**, 3071 (2022).
12. H. Hamada *et al.*, Allele-Specific Methylome and Transcriptome Analysis Reveals Widespread Imprinting in the Human Placenta. *Am J Hum Genet* **99**, 1045-1058 (2016).
13. B. Langmead, S. L. Salzberg, Fast gapped-read alignment with Bowtie 2. *Nat Methods* **9**, 357-359 (2012).
14. Y. Zhang *et al.*, Model-based analysis of ChIP-Seq (MACS). *Genome Biol* **9**, R137 (2008).
15. S. Heinz *et al.*, Simple combinations of lineage-determining transcription factors prime cis-regulatory elements required for macrophage and B cell identities. *Mol Cell* **38**, 576-589 (2010).
16. H. M. Amemiya, A. Kundaje, A. P. Boyle, The ENCODE Blacklist: Identification of Problematic Regions of the Genome. *Sci Rep* **9**, 9354 (2019).
17. M. R. Mumbach *et al.*, HiChIP: efficient and sensitive analysis of protein-directed genome architecture. *Nat Methods* **13**, 919-922 (2016).
18. R. Fang *et al.*, Mapping of long-range chromatin interactions by proximity ligation-assisted ChIP-seq. *Cell Res* **26**, 1345-1348 (2016).
19. B. E. Gryder, J. Khan, B. Z. Stanton, Measurement of differential chromatin interactions with absolute quantification of architecture (AQuA-HiChIP). *Nat Protoc* **15**, 1209-1236 (2020).
20. N. Servant *et al.*, HiC-Pro: an optimized and flexible pipeline for Hi-C data processing. *Genome Biol* **16**, 259 (2015).
21. T. Yang *et al.*, HiCRep: assessing the reproducibility of Hi-C data using a stratum-adjusted correlation coefficient. *Genome Res* **27**, 1939-1949 (2017).
22. S. Bhattacharyya, V. Chandra, P. Vijayanand, F. Ay, Identification of significant chromatin contacts from HiChIP data by FitHiChIP. *Nat Commun* **10**, 4221 (2019).

23. A. Arutyunyan *et al.*, Spatial multiomics map of trophoblast development in early pregnancy. *Nature* **616**, 143-151 (2023).
24. R. Vento-Tormo *et al.*, Single-cell reconstruction of the early maternal-fetal interface in humans. *Nature* **563**, 347-353 (2018).
25. F. Ramirez *et al.*, deepTools2: a next generation web server for deep-sequencing data analysis. *Nucleic Acids Res* **44**, W160-165 (2016).
26. J. T. Robinson *et al.*, Integrative genomics viewer. *Nat Biotechnol* **29**, 24-26 (2011).
27. M. V. Kuleshov *et al.*, Enrichr: a comprehensive gene set enrichment analysis web server 2016 update. *Nucleic Acids Res* **44**, W90-97 (2016).
28. Y. Chen, D. Siriwardena, C. Penfold, A. Pavlinek, T. E. Boroviak, An integrated atlas of human placental development delineates essential regulators of trophoblast stem cells. *Development* **149** (2022).
29. L. M. Ferreira *et al.*, A distant trophoblast-specific enhancer controls HLA-G expression at the maternal-fetal interface. *Proc Natl Acad Sci U S A* **113**, 5364-5369 (2016).
30. D. Strumpf *et al.*, Cdx2 is required for correct cell fate specification and differentiation of trophectoderm in the mouse blastocyst. *Development* **132**, 2093-2102 (2005).
31. M. Donnison *et al.*, Loss of the extraembryonic ectoderm in Elf5 mutants leads to defects in embryonic patterning. *Development* **132**, 2299-2308 (2005).
32. A. P. Russ *et al.*, Eomesodermin is required for mouse trophoblast development and mesoderm formation. *Nature* **404**, 95-99 (2000).
33. J. Luo *et al.*, Placental abnormalities in mouse embryos lacking the orphan nuclear receptor ERR-beta. *Nature* **388**, 778-782 (1997).
34. P. A. Latos *et al.*, Fgf and Esrrb integrate epigenetic and transcriptional networks that regulate self-renewal of trophoblast stem cells. *Nat Commun* **6**, 7776 (2015).
35. D. M. Tompers, R. K. Foreman, Q. Wang, M. Kumanova, P. A. Labosky, Foxd3 is required in the trophoblast progenitor cell lineage of the mouse embryo. *Dev Biol* **285**, 126-137 (2005).
36. X. Yang, C. Li, X. Xu, C. Deng, The tumor suppressor SMAD4/DPC4 is essential for epiblast proliferation and mesoderm induction in mice. *Proc Natl Acad Sci U S A* **95**, 3667-3672 (1998).
37. A. A. Avilion *et al.*, Multipotent cell lineages in early mouse development depend on SOX2 function. *Genes Dev* **17**, 126-140 (2003).
38. H. Yamamoto *et al.*, Defective trophoblast function in mice with a targeted mutation of Ets2. *Genes Dev* **12**, 1315-1326 (1998).
39. F. Wen *et al.*, Ets2 is required for trophoblast stem cell self-renewal. *Dev Biol* **312**, 284-299 (2007).
40. C. Polydorou, P. Georgiades, Ets2-dependent trophoblast signalling is required for gastrulation progression after primitive streak initiation. *Nat Commun* **4**, 1658 (2013).
41. P. Home *et al.*, Genetic redundancy of GATA factors in the extraembryonic trophoblast lineage ensures the progression of preimplantation and postimplantation mammalian development. *Development* **144**, 876-888 (2017).
42. V. Sebastiano *et al.*, Oct1 regulates trophoblast development during early mouse embryogenesis. *Development* **137**, 3551-3560 (2010).
43. B. Saha *et al.*, TEAD4 ensures postimplantation development by promoting trophoblast self-renewal: An implication in early human pregnancy loss. *Proc Natl Acad Sci U S A* **117**, 17864-17875 (2020).

44. R. Yagi *et al.*, Transcription factor TEAD4 specifies the trophectoderm lineage at the beginning of mammalian development. *Development* **134**, 3827-3836 (2007).
45. H. J. Auman *et al.*, Transcription factor AP-2gamma is essential in the extra-embryonic lineages for early postimplantation development. *Development* **129**, 2733-2747 (2002).
46. T. Goller, F. Vauti, S. Ramasamy, H. H. Arnold, Transcriptional regulator BPTF/FAC1 is essential for trophoblast differentiation during early mouse development. *Mol Cell Biol* **28**, 6819-6827 (2008).
47. P. Riley, L. Anson-Cartwright, J. C. Cross, The Hand1 bHLH transcription factor is essential for placentation and cardiac morphogenesis. *Nat Genet* **18**, 271-275 (1998).
48. X. Xiao *et al.*, HSF1 is required for extra-embryonic development, postnatal growth and protection during inflammatory responses in mice. *EMBO J* **18**, 5943-5952 (1999).
49. D. M. Adelman, M. Gertsenstein, A. Nagy, M. C. Simon, E. Maltepe, Placental cell fates are regulated in vivo by HIF-mediated hypoxia responses. *Genes Dev* **14**, 3191-3203 (2000).
50. F. Guillemot, A. Nagy, A. Auerbach, J. Rossant, A. L. Joyner, Essential role of Mash-2 in extraembryonic development. *Nature* **371**, 333-336 (1994).
51. N. C. Dubois *et al.*, Placental rescue reveals a sole requirement for c-Myc in embryonic erythroblast survival and hematopoietic stem cell function. *Development* **135**, 2455-2465 (2008).
52. Y. Barak *et al.*, PPAR gamma is required for placental, cardiac, and adipose tissue development. *Mol Cell* **4**, 585-595 (1999).
53. K. Nadra *et al.*, PPARgamma in placental angiogenesis. *Endocrinology* **151**, 4969-4981 (2010).
54. I. Kruger *et al.*, Sp1/Sp3 compound heterozygous mice are not viable: impaired erythropoiesis and severe placental defects. *Dev Dyn* **236**, 2235-2244 (2007).
55. M. J. Kohn, R. T. Bronson, E. Harlow, N. J. Dyson, L. Yamasaki, Dp1 is required for extra-embryonic development. *Development* **130**, 1295-1305 (2003).
56. V. Begay, J. Smink, A. Leutz, Essential requirement of CCAAT/enhancer binding proteins in embryogenesis. *Mol Cell Biol* **24**, 9744-9751 (2004).
57. M. I. Morasso, A. Grinberg, G. Robinson, T. D. Sargent, K. A. Mahon, Placental failure in mice lacking the homeobox gene Dlx3. *Proc Natl Acad Sci U S A* **96**, 162-167 (1999).
58. C. Papadaki *et al.*, Transcriptional repressor erf determines extraembryonic ectoderm differentiation. *Mol Cell Biol* **27**, 5201-5213 (2007).
59. M. Schreiber *et al.*, Placental vascularisation requires the AP-1 component fra1. *Development* **127**, 4937-4948 (2000).
60. L. Anson-Cartwright *et al.*, The glial cells missing-1 protein is essential for branching morphogenesis in the chorioallantoic placenta. *Nat Genet* **25**, 311-314 (2000).
61. S. Unezaki, R. Horai, K. Sudo, Y. Iwakura, S. Ito, Ovol2/Movo, a homologue of Drosophila ovo, is required for angiogenesis, heart formation and placental development in mice. *Genes Cells* **12**, 773-785 (2007).
62. V. Sapin, P. Dolle, C. Hindelang, P. Kastner, P. Chambon, Defects of the chorioallantoic placenta in mouse RXRalpha null fetuses. *Dev Biol* **191**, 29-41 (1997).
63. E. Steingrimsson, L. Tessarollo, S. W. Reid, N. A. Jenkins, N. G. Copeland, The bHLH-Zip transcription factor Tfeb is essential for placental vascularization. *Development* **125**, 4607-4616 (1998).

64. S. Tanaka, T. Kunath, A. K. Hadjantonakis, A. Nagy, J. Rossant, Promotion of trophoblast stem cell proliferation by FGF4. *Science* **282**, 2072-2075 (1998).
65. E. Arman, R. Haffner-Krausz, Y. Chen, J. K. Heath, P. Lonai, Targeted disruption of fibroblast growth factor (FGF) receptor 2 suggests a role for FGF signaling in pregastrulation mammalian development. *Proc Natl Acad Sci U S A* **95**, 5082-5087 (1998).
66. N. Gotoh *et al.*, The docking protein FRS2alpha is an essential component of multiple fibroblast growth factor responses during early mouse development. *Mol Cell Biol* **25**, 4105-4116 (2005).
67. M. Weinstein *et al.*, Failure of egg cylinder elongation and mesoderm induction in mouse embryos lacking the tumor suppressor smad2. *Proc Natl Acad Sci U S A* **95**, 9378-9383 (1998).
68. M. K. Saba-El-Leil *et al.*, An essential function of the mitogen-activated protein kinase Erk2 in mouse trophoblast development. *EMBO Rep* **4**, 964-968 (2003).
69. M. Sibilia, E. F. Wagner, Strain-dependent epithelial defects in mice lacking the EGF receptor. *Science* **269**, 234-238 (1995).
70. Z. Z. Yang *et al.*, Protein kinase B alpha/Akt1 regulates placental development and fetal growth. *J Biol Chem* **278**, 32124-32131 (2003).
71. G. Galabova-Kovacs *et al.*, Essential role of B-Raf in ERK activation during extraembryonic development. *Proc Natl Acad Sci U S A* **103**, 1325-1330 (2006).
72. J. S. Mudgett *et al.*, Essential role for p38alpha mitogen-activated protein kinase in placental angiogenesis. *Proc Natl Acad Sci U S A* **97**, 10454-10459 (2000).
73. A. W. Roberts *et al.*, Placental defects and embryonic lethality in mice lacking suppressor of cytokine signaling 3. *Proc Natl Acad Sci U S A* **98**, 9324-9329 (2001).
74. J. Lu *et al.*, A positive feedback loop involving Gcm1 and Fzd5 directs chorionic branching morphogenesis in the placenta. *PLoS Biol* **11**, e1001536 (2013).
75. M. Itoh *et al.*, Role of Gab1 in heart, placenta, and skin development and growth factor- and cytokine-induced extracellular signal-regulated kinase mitogen-activated protein kinase activation. *Mol Cell Biol* **20**, 3695-3704 (2000).
76. S. Giroux *et al.*, Embryonic death of Mek1-deficient mice reveals a role for this kinase in angiogenesis in the labyrinthine region of the placenta. *Curr Biol* **9**, 369-372 (1999).
77. M. Sachs *et al.*, Essential role of Gab1 for signaling by the c-Met receptor in vivo. *J Cell Biol* **150**, 1375-1384 (2000).
78. M. Ueno *et al.*, c-Met-dependent multipotent labyrinth trophoblast progenitors establish placental exchange interface. *Dev Cell* **27**, 373-386 (2013).
79. R. Ohlsson *et al.*, PDGFB regulates the development of the labyrinthine layer of the mouse fetal placenta. *Dev Biol* **212**, 124-136 (1999).
80. C. Shiota, J. T. Woo, J. Lindner, K. D. Shelton, M. A. Magnuson, Multiallelic disruption of the rictor gene in mice reveals that mTOR complex 2 is essential for fetal growth and viability. *Dev Cell* **11**, 583-589 (2006).
81. T. A. Rodriguez *et al.*, Cited1 is required in trophoblasts for placental development and for embryo growth and survival. *Mol Cell Biol* **24**, 228-244 (2004).
82. Y. Li, R. R. Behringer, Esx1 is an X-chromosome-imprinted regulator of placental development and fetal growth. *Nat Genet* **20**, 309-311 (1998).

83. K. Denda *et al.*, Nrk, an X-linked protein kinase in the germinal center kinase family, is required for placental development and fetoplacental induction of labor. *J Biol Chem* **286**, 28802-28810 (2011).
84. S. M. Jackman, X. Kong, M. E. Fant, Plac1 (placenta-specific 1) is essential for normal placental and embryonic development. *Mol Reprod Dev* **79**, 564-572 (2012).
85. K. Takahashi, T. Kobayashi, N. Kanayama, p57(Kip2) regulates the proper development of labyrinthine and spongiotrophoblasts. *Mol Hum Reprod* **6**, 1019-1025 (2000).
86. C. DeRossi *et al.*, Ablation of mouse phosphomannose isomerase (Mpi) causes mannose 6-phosphate accumulation, toxicity, and embryonic lethality. *J Biol Chem* **281**, 5916-5927 (2006).
87. A. Suzuki *et al.*, High cancer susceptibility and embryonic lethality associated with mutation of the PTEN tumor suppressor gene in mice. *Curr Biol* **8**, 1169-1178 (1998).
88. M. Charalambous *et al.*, Maternally-inherited Grb10 reduces placental size and efficiency. *Dev Biol* **337**, 1-8 (2010).
89. D. Frank *et al.*, Placental overgrowth in mice lacking the imprinted gene Ipl. *Proc Natl Acad Sci U S A* **99**, 7490-7495 (2002).
90. L. Wu *et al.*, Extra-embryonic function of Rb is essential for embryonic development and viability. *Nature* **421**, 942-947 (2003).
91. K. D. Tremblay, N. R. Dunn, E. J. Robertson, Mouse embryos lacking Smad1 signals display defects in extra-embryonic tissues and germ cell formation. *Development* **128**, 3609-3621 (2001).
92. M. Li *et al.*, The adaptor protein of the anaphase promoting complex Cdh1 is essential in maintaining replicative lifespan and in learning and memory. *Nat Cell Biol* **10**, 1083-1089 (2008).
93. I. Garcia-Higuera *et al.*, Genomic stability and tumour suppression by the APC/C cofactor Cdh1. *Nat Cell Biol* **10**, 802-811 (2008).
94. D. Jaquemar *et al.*, Keratin 8 protection of placental barrier function. *J Cell Biol* **161**, 749-756 (2003).
95. N. Kraut, L. Snider, C. M. Chen, S. J. Tapscott, M. Groudine, Requirement of the mouse I-mfa gene for placental development and skeletal patterning. *EMBO J* **17**, 6276-6288 (1998).
96. A. Mould, M. A. Morgan, L. Li, E. K. Bikoff, E. J. Robertson, Blimp1/Prdm1 governs terminal differentiation of endovascular trophoblast giant cells and defines multipotent progenitors in the developing placenta. *Genes Dev* **26**, 2063-2074 (2012).

Supplemental Figures and Tables

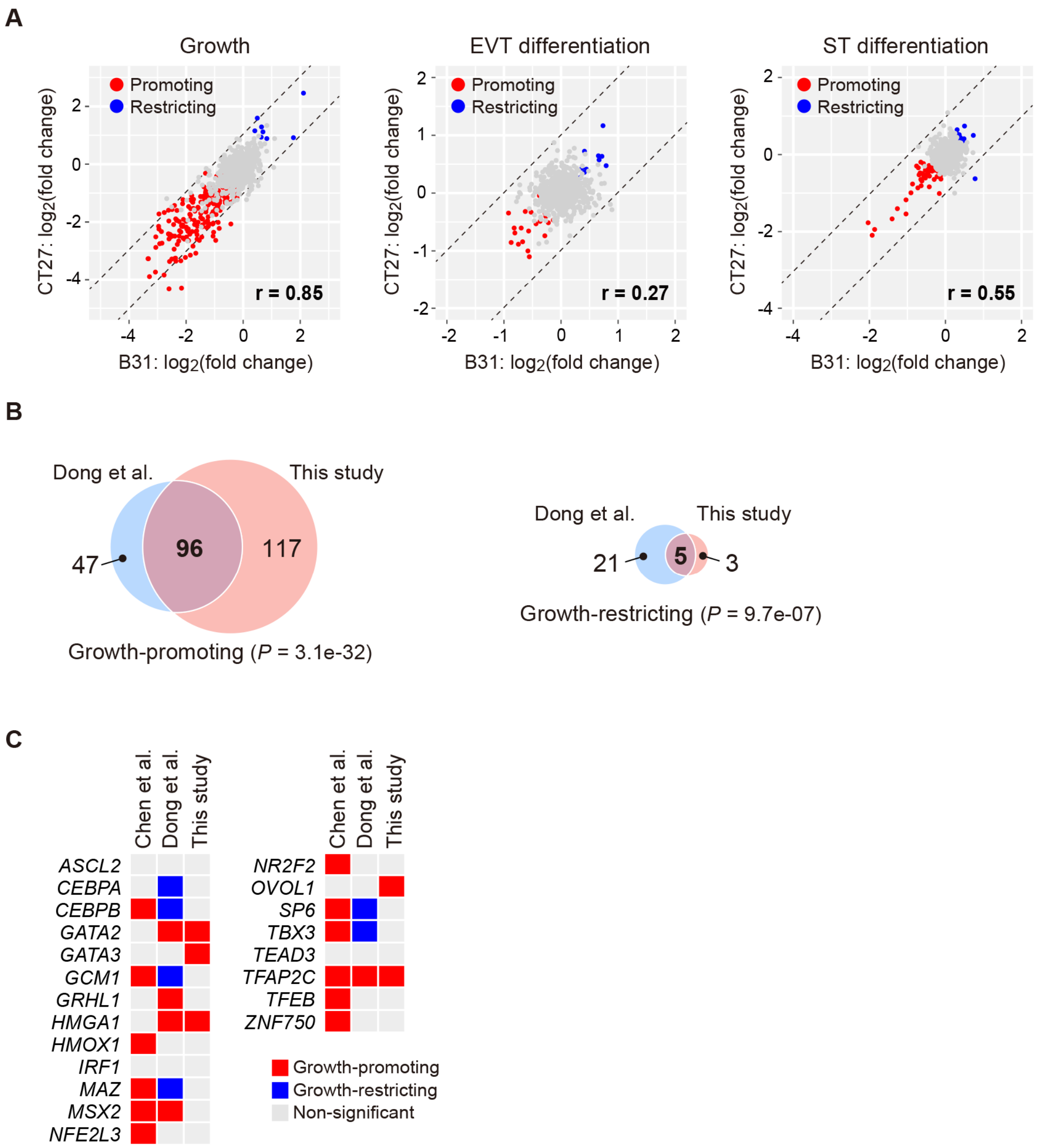

Figure S1. Validation of CRISPR screening. (**A**) Reproducibility of CRISPR screening. Two hTSC lines, CT27 and B31, were subjected to CRISPR screening for regulators of hTSC growth (left), EVT differentiation (middle), and ST differentiation (right). Fold-changes in normalized gRNA read counts per gene were calculated using MAGeCK (6). The x-axis indicates log_2(fold change) for B31, and the y-axis indicates log_2(fold change) for CT27. The absolute differences in log_2(fold change) between CT27 and B31 were less than 1 for most of the analyzed genes (those within the dotted lines), suggesting high reproducibility between the biological duplicates. Genes that promote hTSC growth or differentiation are shown in red, and those that restrict hTSC growth or differentiation are in blue. The Pearson correlation coefficients (r) are indicated. Note that the Pearson correlation coefficients were not high for the screening of EVT (r = 0.27) and ST regulators (r = 0.55), which is not due to poor reproducibility, but because most of the analyzed

genes were classified as non-significant. (**B**) Comparison of the results of CRISPR screening in this study and from Dong et al. (5). Only the 850 genes in Fig. 1A were considered. The left figure shows growth-promoting genes, and the right shows growth-restricting genes. *P*-values were calculated using Fisher's exact test. (**C**) Comparison of the results of RNAi screening by Chen et al.(28) with those of CRISPR screening by us and Dong et al. (5). Chen et al. performed RNAi screening targeting 24 genes in hTSCs. Among these, 21 genes were included in our screening and are listed in this figure. Genes that promote hTSC growth are shown in red, and those that restrict hTSC growth are in blue.

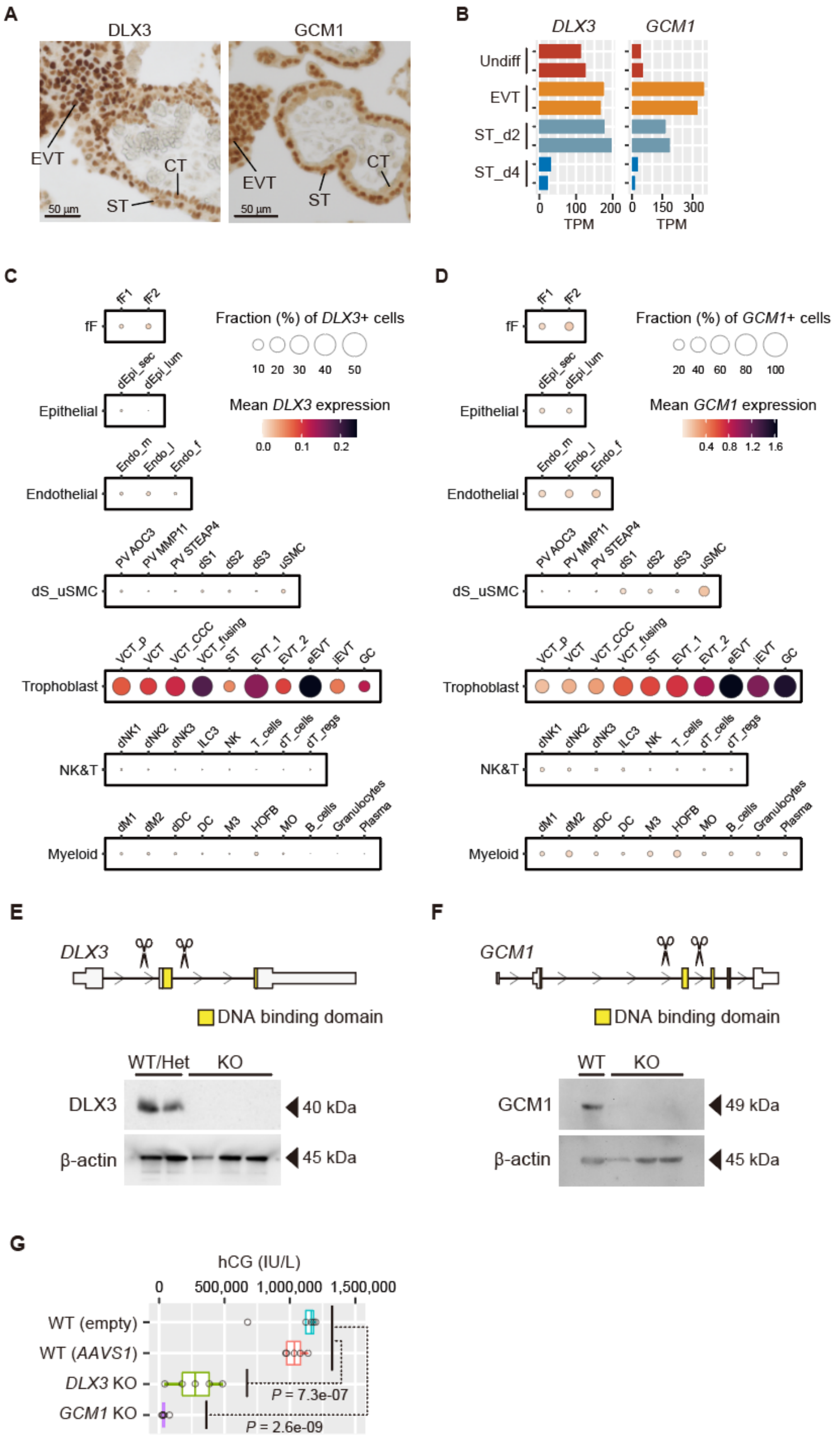
A
DLX3
GCM1
EVT
CT
ST
50 µm
B
DLX3
GCM1
Undiff
EVT
ST_d2
ST_d4
0 100 200
0 150 300
TPM
C
fF
fF1
fF2
Fraction (%) of DLX3+ cells
10 20 30 40 50
Epithelial
dEpi_sec
dEpi_lum
Mean DLX3 expression
0.0 0.1 0.2
Endothelial
Endo_m
Endo_l
Endo_f
dS_uSMC
PV AOC3
PV MMP11
PV STEAP4
dS1
dS2
dS3
uSMC
Trophoblast
VCT_p
VCT
VCT_CCC
VCT_fusing
ST
EVT_1
EVT_2
eEVT
iEVT
GC
NK&T
dNK1
dNK2
dNK3
ILC3
NK
T_cells
dT_cells
dT_regs
Myeloid
dM1
dM2
dDC
DC
M3
HOFB
MO
B_cells
Granulocytes
Plasma
D
Fraction (%) of GCM1+ cells
20 40 60 80 100
Mean GCM1 expression
0.4 0.8 1.2 1.6
E
DLX3
DNA binding domain
WT/Het
KO
DLX3
40 kDa
β-actin
45 kDa
F
GCM1
DNA binding domain
WT
KO
GCM1
49 kDa
β-actin
45 kDa
G
hCG (IU/L)
0 500,000 1,000,000 1,500,000
WT (empty)
WT (AAVS1)
DLX3 KO
GCM1 KO
P = 7.3e-07
P = 2.6e-09

Figure S2. Expression patterns of *DLX3* and *GCM1* and generation of their KO TSCs. (**A**) Immunostaining of first-trimester human placentas. DLX3 was detected in the nuclei of all trophoblast lineages. GCM1 was detected in the nuclei of EVTs and STs. Representative images of two technical replicates are shown. (**B**) RNA-Seq analysis of undifferentiated (Undiff) and differentiated hTSCs. Gene expression levels are expressed as TPM values. ST_d2: STs two days after differentiation induction; ST_d4: STs four days after differentiation induction. (**C**) Dot plots showing log-transformed, normalized expression levels (color) and proportion of cells expressing *DLX3* (dot size). scRNA-Seq and snRNA-Seq data for first-trimester human placentas, including cell type annotations, were obtained from previous studies (23, 24). *DLX3* was highly expressed in villous cytotrophoblasts (VCT), VCT differentiating into STs (VCT_fusing), and EVTs. Fetal (f), fibroblasts (F), decidual (d), epithelial (Epi), secretory (sec), luminal (lum), endothelial (Endo), maternal (m), lymphatic (l), perivascular cells (PV), stromal (S), uterine smooth muscle cells (uSMC), proliferative (p), cytotrophoblast cell column (CCC), endovascular EVT (eEVT), interstitial EVT (iEVT), giant cells (GC), natural killer (NK), innate lymphocytes (ILC), macrophages (M), dendritic cells (DC), Hofbauer cells (HOFB), and monocytes (MO). (**D**) Dot plots showing log-transformed, normalized expression levels and proportion of cells expressing *GCM1*. *GCM1* was highly expressed in VCT_fusing, ST, and EVTs. Data are presented as in (C). (**E**) Generation of *DLX3* KO hTSCs. Exon 2 of *DLX3*, which contains most of the DNA binding domain, was deleted using CRISPR/Cas9. The absence of DLX3 in the *DLX3* KO clones was confirmed by western blotting. (**F**) Generation of *GCM1* KO hTSCs. Exon 3 of *GCM1*, which contains most of the DNA binding domain, was deleted using CRISPR/Cas9. The absence of GCM1 in the *GCM1* KO clones was confirmed by western blotting. (**G**) Quantification of hCG secreted by STs differentiated from *DLX3* KO and *GCM1* KO clones. hCG secretion was quantified using ELISA. Five independent clones were analyzed for each genotype. *P*-values were calculated using the Student’s t-test.

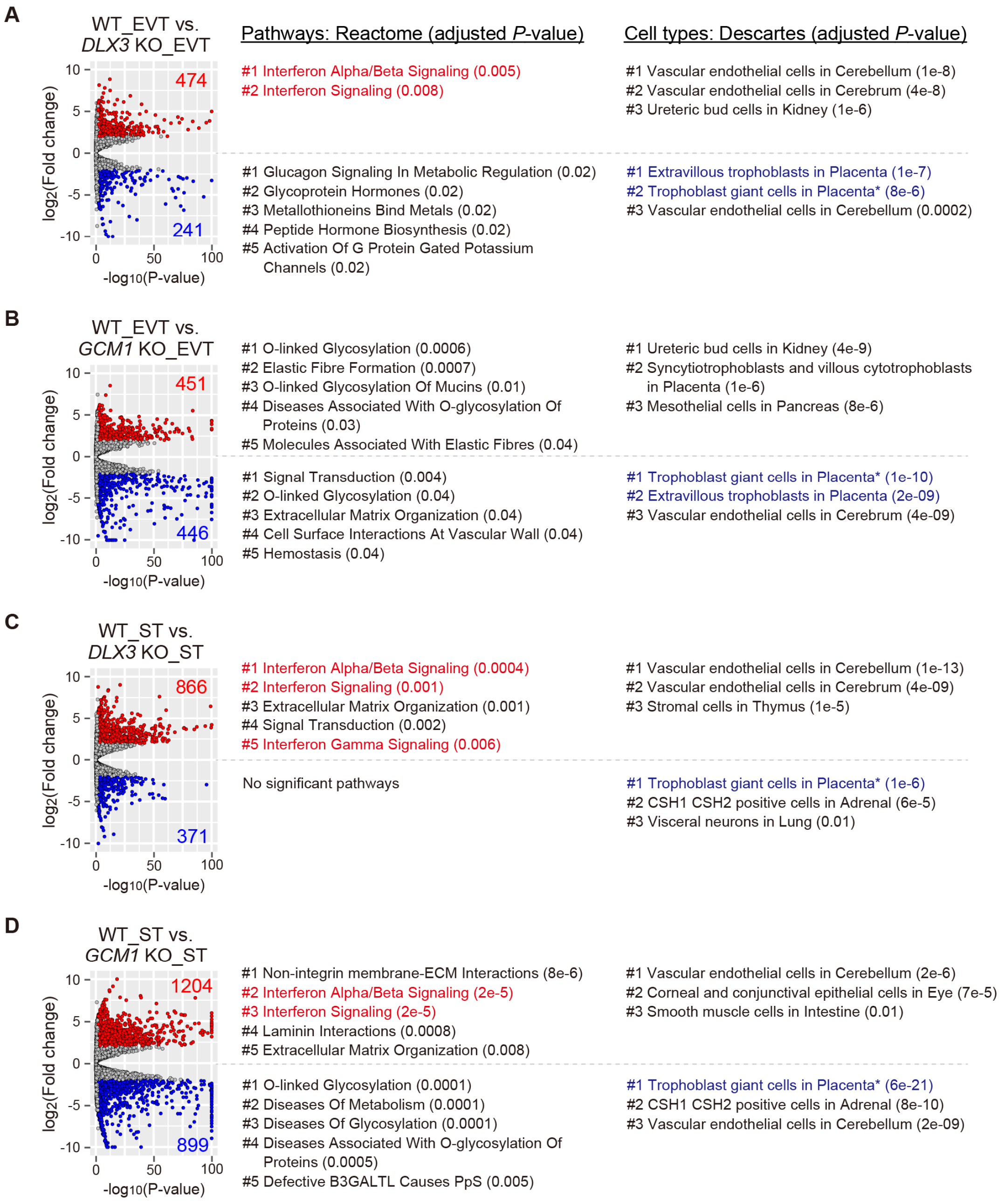

Figure S3. Enrichment analysis of DEGs between WT and *DLX3* KO clones and between WT and *GCM1* KO clones. (**A**) Analysis of DEGs between WT and *DLX3* KO EVTs. Up- and down-regulated genes in *DLX3* KO EVTs (adjusted *P*-value < 0.05, fold change > 2) are shown in red and blue, respectively. We analyzed the enriched pathways (based on the Reactome dataset) and cell types (based on the Descartes dataset) using Enrichr (27). Only the top five pathways and top three cell types with adjusted *P*-values <0.05 are indicated. The pathway enrichment analysis revealed that pathways associated with interferon responses were up-regulated in *DLX3* KO EVTs (red). The cell type enrichment analysis showed that EVT and ST markers were down-regulated in *DLX3* KO EVTs (blue). *Note that STs are classified as “Trophoblast giant cells in Placenta” in the Descartes dataset. (**B**) Analysis of DEGs between WT and *GCM1* KO EVTs.

Enriched pathways and cell types were identified as in (A). EVT and ST markers were down-regulated in *GCM1* KO EVTs (blue). (**C**) Analysis of DEGs between WT and *DLX3* KO STs. Enriched pathways and cell types were identified as in (A). Pathways associated with interferon responses were up-regulated in *DLX3* KO STs (red). ST markers were down-regulated in *DLX3* KO STs (blue). (**D**) Analysis of DEGs between WT and *GCM1* KO STs. Enriched pathways and cell types were identified as in (A). Pathways associated with interferon responses were up-regulated in *GCM1* KO STs (red). ST markers were down-regulated in *DLX3* KO STs (blue).

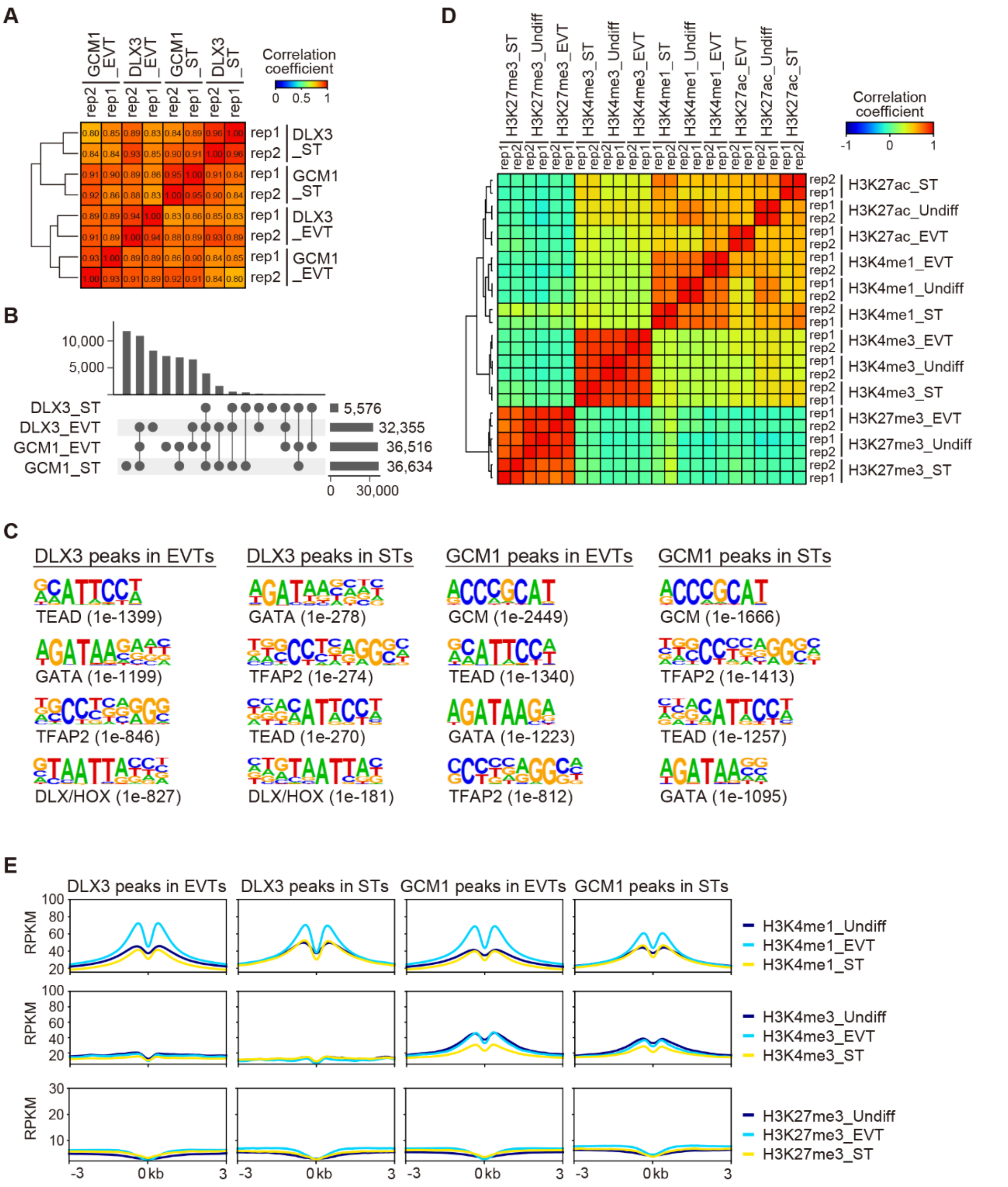

Figure S4. Identification of DLX3 and GCM1 binding peaks and their association with histone modifications. (**A**) ChIP-Seq of DLX3 and GCM1 in EVTs and STs. Two independent hTSC lines were analyzed; their data are presented as replicates 1 and 2 (rep 1 and 2). Pearson correlation coefficients between ChIP-Seq data (bin size = 10 kb) were calculated. (**B**) Upset plot of ChIP-Seq peaks of DLX3 and GCM1. Only peaks shared between biological replicates were analyzed. (**C**) Enriched motifs in the DLX3 and GCM1 ChIP-Seq peaks. The top four enriched motifs are indicated with their *P*-values and best-matched known TFs. (**D**) ChIP-Seq of histone modifications in undifferentiated hTSCs (Undiff), EVTs, and STs. Two independent hTSC lines were analyzed, and Pearson correlation coefficients between ChIP-Seq data (bin size = 10 kb) were calculated.

(**E**) Histone modification patterns around DLX3 and GCM1 peaks. DLX3 and GCM1 binding sites were identified in both EVTs and STs. ChIP-Seq of H3K4me1, H3K4me3, and H3K27me3 was performed in undifferentiated hTSCs, EVTs, and STs. Averaged H3K4me1, H3K4me3, and H3K27me3 signals around DLX3 and GCM1 peaks are expressed as RPKM. Differentiation-dependent changes in signal intensities were less obvious for H3K4me1 compared to those for H3K27ac (Fig. 3B) and barely detectable for H3K4me3 and H3K27me3.

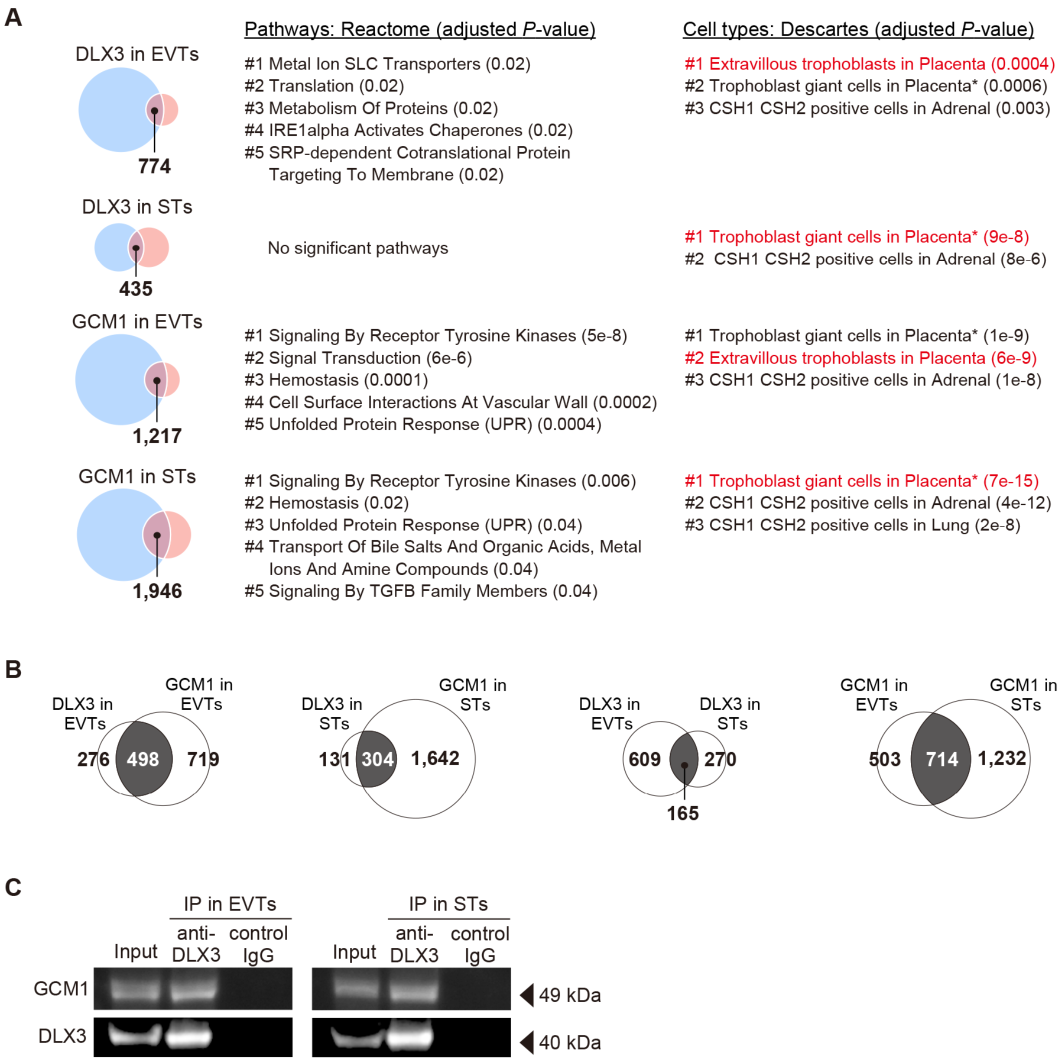

Figure S5. Characterization of DLX3- and GCM1-binding peaks. (**A**) Enriched pathways and cell types among potential target genes of DLX3 and GCM1. Potential target genes of DLX3 and GCM1 were identified by merging the genes near their ChIP-Seq peaks with the genes down-regulated by their respective KO (see Fig. 3C). Enriched pathways (based on the Reactome dataset) and cell types (based on the Descartes dataset) were identified using Enrichr (27). Cell type enrichment analysis revealed that ST and/or EVT markers were enriched among the potential target genes of DLX3 and GCM1 (red). *Note that STs are classified as “Trophoblast giant cells in Placenta” in the Descartes dataset. (**B**) Overlaps between the potential target genes of DLX3 and GCM1. The two figures on the left show that the majority of the potential targets of DLX3 were also targeted by GCM1 in both EVTs and STs. The two figures on the right reveal that DLX3 and GCM1 exhibited both overlapping and distinct potential target genes between EVTs and STs. (**C**) Co-IP of GCM1 with DLX3. EVTs (left) and STs (right) derived from hTSCs were subjected to IP using an anti-DLX3 antibody or control IgG and were analyzed using western blotting.

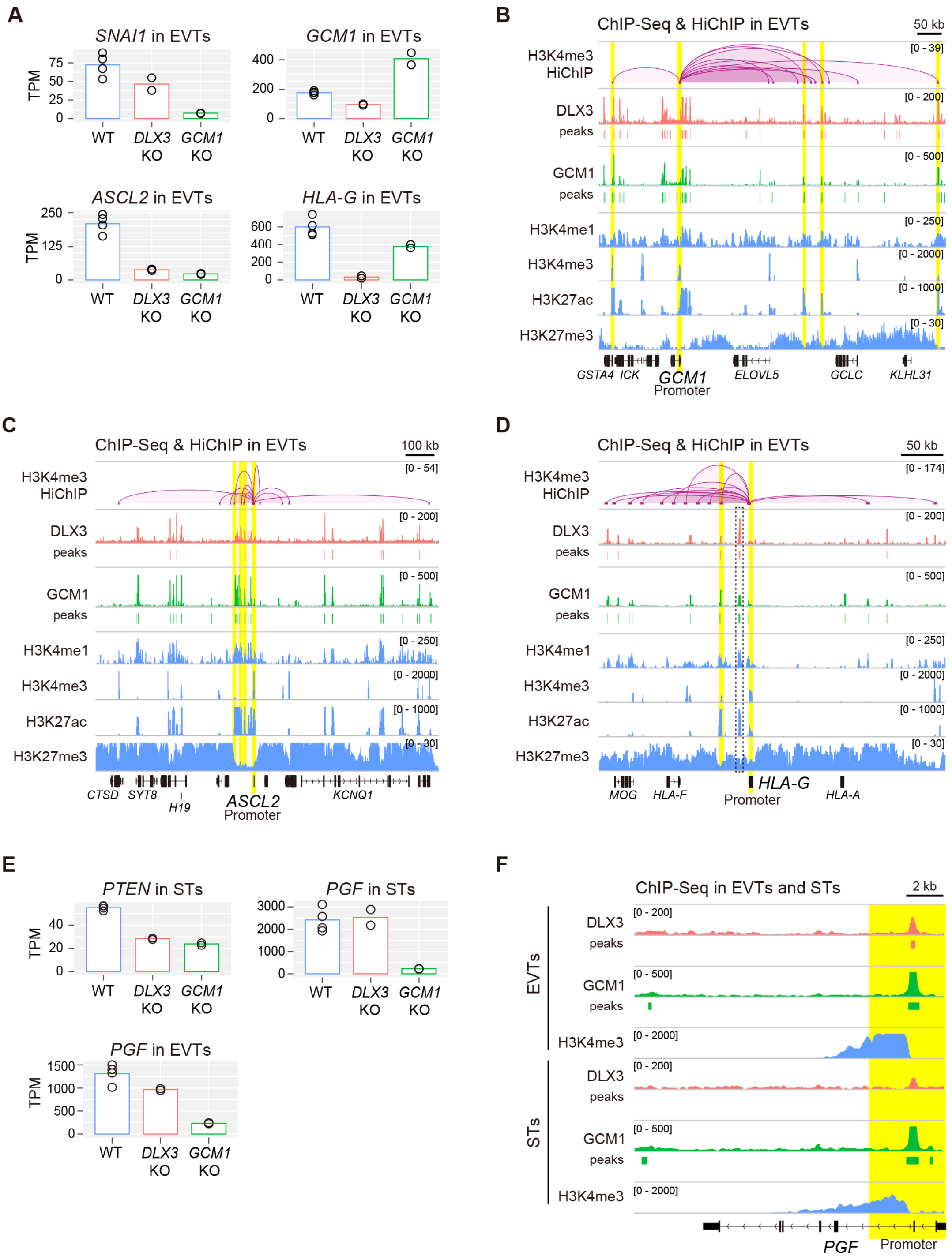

Figure S6. Examples of interactions of DLX3 and GCM1 peaks with their potential target genes. (**A**) Expression levels of selected genes in EVTs derived from four WT, two *DLX3* KO, and two *GCM1* KO clones. Gene expression levels were analyzed via RNA-Seq and are expressed as TPM. (**B**) Analysis of the *GCM1* locus. The data were obtained in EVTs. The H3K4me3-marked

GCM1 promoter is indicated in yellow. H3K27ac-marked enhancers containing DLX3 peaks and which physically interacted with the *SNAI1* promoter are also shown in yellow. The y-axis indicates RPKM for the ChIP-Seq data and $-\log_{10}$(q-value) for the HiChIP data. (**C**) Analysis of the *ASCL2* locus. The data were obtained in EVTs. (**D**) Analysis of the *HLA-G* locus. The data were obtained in EVTs. An enhancer with DLX3 and GCM1 peaks physically interacted with the *HLA-G* promoter. Additionally, DLX3 and GCM1 peaks were also identified in a known *HLA-G* enhancer (dotted box) (29). Note that only significant long-range (>20 kb) chromatin interactions were considered in this study, whereas the distance between the *HLA-G* promoter and the known *HLA-G* enhancer is less than 20 kb. (**E**) Expression levels of *PTEN* and *PGF* in STs or EVTs derived from four WT, two *DLX3* KO, and two *GCM1* KO clones. (**F**) Analysis of the *PGF* locus. The data were obtained in EVTs and STs. DLX3 bound the transcription start site of *PGF* in EVTs. GCM1 bound the transcription start site of *PGF* in both EVTs and STs.

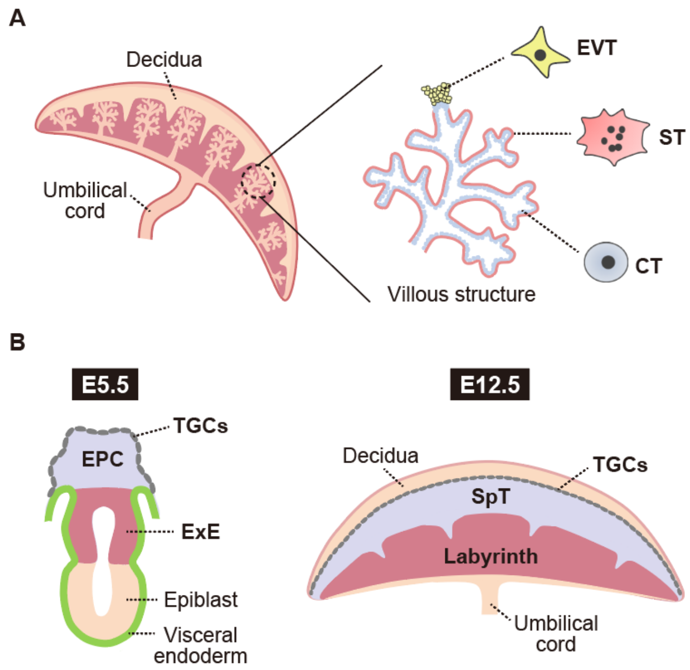

Figure S7. Schematic representation of human and mouse placentas. (**A**) Anatomy of the human placenta. The placental villi are covered by two layers of trophoblasts, CTs and STs. CTs function as progenitor cells of STs and EVTs. STs are formed by the fusion of CTs. At the tips of some villi, CTs differentiate into EVTs. The EVT subtypes and non-trophoblast cells are omitted. (**B**) Anatomy of embryonic day 5.5 (E5.5) and E12.5 mouse placentas. TE gives rise to ExE, EPC, and TGCs after implantation (left). ExE further differentiates into the chorion, and the chorion fuses to the allantois to form the labyrinth layer. EPC contributes to SpT and TGCs (right). The TGC subtypes are omitted.

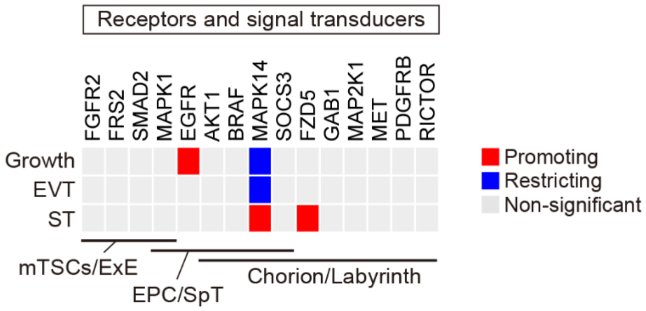

Figure S8. Analysis of growth factor receptors and their downstream signal transducers involved in the development of mTSCs/ExE, EPC/SpT, or chorion/labyrinth in mice. Genes promoting hTSC growth or differentiation are shown in red, genes restricting hTSC growth or differentiation are in blue, and those without significant effect are in grey.

Dataset S1. Read mapping summary and count data of CRISPR screening. Two sgRNA libraries were used, one targeting TFs and the other targeting genes essential for mouse placental development. Both libraries contained negative controls (sgRNA#1833–1882 and sgRNA#1573–1672, respectively).

Dataset S2. Summary of CRISPR screening results. Summarized results of CRISPR screening for hTSC growth, EVT differentiation, and ST differentiation regulators. Two sgRNA libraries were used, one targeting TFs and the other targeting genes essential for mouse placental development.

Dataset S3. Growth-promoting and -restricting genes identified in both our study and the CRISPR screening by Dong et al. (5).

Dataset S4. Summary of RNA-Seq data. Read-count data and TPM values are presented. Two hTSC lines, CT27 and B31, were analyzed.

Dataset S5. Predicted target genes of DLX3 and GCM1. To predict the target genes of DLX3 and GCM1, we merged the genes near their ChIP-Seq peaks with those downregulated by their respective KO (Fig. 3C). The resultant genes are listed as predicted target genes.

Dataset S6. Oligonucleotides used for vector construction and PCR.

Dataset S7. Summary of HiChIP data. The amount of DNA used for immunoprecipitation and the number of valid pairs are shown. The samples and spike-in reads were mapped to hg38 and mm10, respectively.

Table S1. Placental phenotypes of KO mice mentioned in Figs. 4 and S8.

Gene symbol	Brief description of placental phenotypes and gene expression patterns	Associated figure	Reference
Cdx2	*Cdx2* is essential for the segregation of the ICM and TE lineages, and mutant embryos die around implantation. In E5.5 embryos, *Cdx2* is expressed in the ExE, but not in the EPC.	Fig. 4C	(30)
Elf5	Mutant embryos lack the ExE, and mTSCs cannot be derived from these embryos. In E5.5 embryos, *Elf5* is expressed in the ExE, but not in the EPC.	Fig. 4C	(31)
Eomes	Mutant embryos arrest at the blastocyst stage. *Eomes* may be required for the development of mTSCs. In E5.0 embryos, *Eomes* is expressed in the ExE, but not in the EPC.	Fig. 4C	(32)
Esrrb	Mutant embryos show abnormal chorion development. *Esrrb* is essential for the maintenance of mTSCs. In E5.5 embryos, *Esrrb* is expressed in the ExE, but not in the EPC.	Fig. 4C	(33, 34)
Foxd3	Mutant embryos do not have self-renewing trophoblast progenitors, and mTSCs cannot be derived from these embryos. FOXD3 is expressed in mTSCs. In E6.5 embryos, FOXD3 is predominantly expressed in the epiblast.	Fig. 4C	(35)
Smad4	The ExE is reduced or absent in mutant embryos. The size of the EPC is proportional to that of the mutant embryos. *Smad4* is expressed ubiquitously.	Fig. 4C	(36)

Sox2	Mutant embryos fail to survive shortly after implantation, and mTSCs cannot be derived from these embryos. In E6.5 embryos, *Sox2* is expressed in the ExE, but not in the EPC.	Fig. 4C	(37)
Ets2	The chorion is not formed and the EPC is small in mutant embryos. ETS2 maintains the expression of *Elf5*, *Cdx2*, and *Eomes in vivo*, and is essential for the maintenance of mTSCs. In E6 embryos, *Ets2* is expressed in the ExE and EPC.	Fig. 4C	(38-40)
Gata2/3	The ExE and EPC are not properly developed in *Gata2/3* double KO embryos. Concomitant loss of *Gata2* and *Gata3* induces mTSC differentiation. GATA2 and GATA3 expression is detectable in the TE and trophoblasts of E7.25 embryos.	Fig. 4C	(41)
Pou2f1	The ExE is severely compromised and the EPC is not formed in mutant embryos. mTSCs cannot be derived from these embryos. POU2F1 is ubiquitously expressed.	Fig. 4C	(42)
Tead4	TE specification is compromised in mutant embryos, and mTSCs cannot be derived from these embryos. Conditional deletion of *Tead4* causes sever defects in the ExE and EPC. TEAD4 is abundantly expressed in the ExE and EPC.	Fig. 4C	(43, 44)
Tfap2c	Mutant embryos exhibit disorganized ExE and small EPC. *Tfap2c* is detectable in the ExE, EPC, and TGCs.	Fig. 4C	(45)
Bptf	The EPC is dramatically reduced in mutant embryos. *Bptf* is expressed in the ExE and EPC. *Bptf* mutation does not impair the TSC pool in the ExE.	Fig. 4C	(46)
Hand1	The EPC is about 20% of its normal size, and TGC differentiation is significantly affected in E7.5 mutant embryos. *Hand1* is expressed in the EPC and TGCs. Note that *Hand1* is not included in Fig. 4F since it is essential for both EPC and TGC development.	Fig. 4C	(47)
Hsf1	The SpT layer is severely reduced in mutant embryos, while the labyrinth layer is histomorphologically normal. HSF1 expression is widespread.	Fig. 4C	(48)
Arnt	The vascularization of the labyrinth layer is severely impaired and the SpT layer is reduced in mutant embryos. ARNT is ubiquitously expressed.	Fig. 4C	(49)
Ascl2	The SpT layer is completely missing and the chorionic ectoderm is reduced in mutant embryos. *Ascl2* is highly expressed in the EPC and chorion.	Fig. 4C	(50)
Myc	Trophoblast cells are severely reduced in number in mutant embryos. MYC is expressed in all compartments of the chorioallantoic placenta.	Fig. 4C	(51)
Pparg	Mutant embryos exhibit abnormal vascularization of the labyrinth layer and decreased SpT cells. *Pparg* is expressed in the EPC and chorion.	Fig. 4C	(52, 53)

Sp1	The SpT layer is small and the labyrinth layer is disorganized in *Sp1*/*Sp3* compound heterozygous mutants. *Sp1* KO mice die around E10.5. *Sp1* is ubiquitously expressed.	Fig. 4C	(54)
Sp3	The SpT layer is small and the labyrinth layer is disorganized in mutant embryos. *Sp3* is ubiquitously expressed.	Fig. 4C	(54)
Tfdp1	Mutant embryos have fewer trophoblasts in the EPC and chorion. TFDP1 is expressed in the EPC, chorion, and TGCs.	Fig. 4C	(55)
Cebpa/b	*Cebpa* and *Cebpb* double KO embryos exhibit impaired vascularization and development of the labyrinth layer. *Cebpa* expression is restricted to the chorion whereas *Cebpb* is expressed in the EPC and chorion.	Fig. 4C	(56)
Dlx3	Vascularization in the labyrinth layer is less extensive in mutant embryos. SpT makers are normally expressed in E9.5 mutant embryos. *Dlx3* is expressed in the EPC and chorion.	Fig. 4C	(57)
Erf	Chorionic cell differentiation is blocked before chorioallantoic attachment and the SpT layer is diminished in mutant embryos. In E8.5 placentas, *Erf* expression is restricted to the chorion, suggesting that the SpT abnormalities may be secondary.	Fig. 4C	(58)
Fosl1	The labyrinth layer is reduced in size and largely avascular in mutant embryos, while the SpT layer is unaffected in these embryos. *Fosl1* is specifically expressed in the labyrinth layer.	Fig. 4C	(59)
Gcm1	The labyrinth layer is not formed owing to the complete block of branching of the chorioallantoic interface in mutant embryos. *Gcm1* is expressed in a subset of trophoblasts in the chorionic plate.	Fig. 4C	(60)
Ovol2	Allantoic blood vessel expansion and development of the labyrinth layer are impaired in mutant embryos. *Ovol2* is expressed in the chorion and labyrinth.	Fig. 4C	(61)
Rxra	The labyrinth layer is disorganized and the SpT layer invades abnormally the labyrinth layer in mutant embryos. RXRα is detected in the labyrinth and TGCs.	Fig. 4C	(62)
Tfeb	Mutant embryos show severe defects in the vascularization of the labyrinth layer. *Tfeb* is highly expressed in the labyrinth layer.	Fig. 4C	(63)
Fgfr2	Mutant embryos die soon after implantation. FGF4 may promote mTSC proliferation via FGFR2. *Fgfr2* is expressed in the ExE.	Fig. S8	(64, 65)
Frs2	Mutant embryos die by E8, and mTSCs cannot be derived from these embryos. FRS2-mediated FGF4 signaling may be required for mTSC maintenance. *Frs2* expression is particularly strong in the ExE.	Fig. S8	(66)

Smad2	Mutant embryos lack the ExE while the EPC appears normal in these embryos. *Smad2* is expressed ubiquitously.	Fig. S8	(67)
Mapk1	Mutant embryos fail to form the ExE and EPC. *Mapk1* is widely expressed at E6.5.	Fig. S8	(68)
Egfr	The SpT layer is reduced in size in mutant embryos. EGFR can be detected in the TE and continues to be expressed in the placenta.	Fig. S8	(69)
Akt1	Mutant embryos show a marked reduction of glycogen trophoblasts in the SpT layer. The labyrinth layer is less vascularized in these embryos. *Akt1* is detected in all placental cell types except decidua.	Fig. S8	(70)
Braf	The SpT and labyrinth layers are severely underdeveloped and disorganized in mutant embryos. B-Raf is expressed in the placenta.	Fig. S8	(71)
Mapk14	Vascularization is severely decreased in the labyrinth layer, and the SpT layer is greatly reduced in size in mutant embryos. Its protein levels are high in diploid trophoblasts.	Fig. S8	(72)
Socs3	The SpT layer is markedly decreased, and the vasculature network in the labyrinth layer is poorly developed in mutant embryos. *Socs3* is expressed in the placenta, including the labyrinth layer.	Fig. S8	(73)
Fzd5	Branching morphogenesis in the chorion fails to occur in mutant embryos. *Fzd5* is mainly detected in trophoblasts of the chorion.	Fig. S8	(74)
Gab1	Trophoblast cells in the labyrinth layer is severely reduced in mutant embryos. The SpT layer is not affected in these embryos. *Gab1* is expressed in both the SpT and labyrinth layers.	Fig. S8	(75)
Map2k1	Vascular endothelial cells in the labyrinth layer are markedly decreased in mutant embryos. *Map2k1* is widely expressed in embryonic and extraembryonic tissues.	Fig. S8	(76)
Met	The size of the labyrinth layer is reduced in mutant embryos. The SpT layer is normal in these embryos. Met is expressed in labyrinth trophoblasts.	Fig. S8	(77, 78)
Pdgfrb	The number of trophoblasts in the labyrinth layer is reduced by about 40% in mutant embryos. *Pdgfrb* expression is restricted to the labyrinth layer.	Fig. S8	(79)
Rictor	The labyrinth layer of mutant embryos is dense and exhibits fewer maternal-fetal interfaces. *Rictor* is ubiquitously expressed.	Fig. S8	(80)
Cited1	The SpT layer is enlarged but the labyrinth layer is reduced in size in mutant embryos. *Cited1* is expressed in all trophoblast lineages.	Fig. 4D	(81)
Esx1	The SpT layer expands and invades into the labyrinth layer in mutant embryos. *Esx1* expression is detected in extraembryonic tissues.	Fig. 4D	(82)

Nrk	The SpT layer is large and invades into the labyrinth layer. *Nrk* is specifically expressed in the SpT layer.	Fig. 4D	(83)
Plac1	Mutant embryos exhibit expanded SpT layer that invades into the labyrinth layer. *Plac1* is expressed in the labyrinth and SpT layers.	Fig. 4D	(84)
Cdkn1c	The numbers of cells in the SpT and labyrinth layers are significantly increased in mutant embryos. *Cdkn1c* is expressed in the labyrinth and SpT layers.	Fig. 4D	(85)
Mpi	The SpT layer, labyrinth layer, and TGCs are hypertrophic in mutant embryos. *Mpi* is ubiquitously expressed.	Fig. 4D	(86)
Pten	Trophoblast cells in the chorion and EPC expand in mutant embryos. PTEN is ubiquitously expressed.	Fig. 4D	(87)
Grb10	The volume of the labyrinth layer is increased in mutant embryos. *Grb10* expression is restricted to the labyrinth layer in the placenta.	Fig. 4D	(88)
Phlda2	The labyrinth layer is also slightly expanded in these embryos. The SpT layer is dramatically expanded in mutant embryos. PHLDA2 expression is restricted to the labyrinth layer.	Fig. 4D	(89)
Rb1	The labyrinth layer of mutant embryos is disrupted with an accumulation of large clusters of densely packed trophoblasts, which may be caused by trophoblast cell hyperproliferation. *Rb1* is expressed in the entire placenta.	Fig. 4D	(90)
Smad1	The chorion over-proliferation is observed in mutant embryos. *Smad1* is expressed in the chorion.	Fig. 4D	(91)
Fzr1	The labyrinth layer is significantly decreased and TGCs are almost absent in mutant embryos. The SpT layer has not been well investigated in these embryos. *Fzr1* is widely expressed.	Fig. 4F	(92, 93)
Krt8	Mutant embryos exhibit the disruption of the TGC layer, which leads to hematomas. The overall structure of the placenta is normal in mutant embryos. *Krt8* is expressed in the labyrinth layer, SpT layer, and TGCs.	Fig. 4F	(94)
Mdfi	The number of TGCs is strongly decreased in mutant embryos. The SpT layer is normal in these embryos. Labyrinth trophoblasts are slightly reduced in some mutant embryos. *Mdfi* is detected in the chorion, EPC, and TGCs.	Fig. 4F	(95)
Prdm1	The specification of spiral artery TGCs is disrupted in mutant embryos. PRDM1 is expressed in spiral artery TGCs and glycogen trophoblasts.	Fig. 4F	(96)
